# Cytometric analysis reveals an association between allergen-responsive natural killer cells and human peanut allergy

**DOI:** 10.1172/JCI157962

**Published:** 2022-10-17

**Authors:** Xiaoying Zhou, Wong Yu, Diane M. Dunham, Jackson P. Schuetz, Catherine A. Blish, Rosemarie H. DeKruyff, Kari C. Nadeau

**Affiliations:** 1 Sean N. Parker Center for Allergy and Asthma Research at Stanford University and Division of Pulmonary, Allergy, and Critical Care Medicine, Stanford, California, USA.; 2 Division of Allergy, Immunology and Blood and Marrow Transplantation, Department of Pediatrics, University of California, San Francisco, California, USA.; 3 Program in Immunology and Department of Medicine, Stanford University School of Medicine, Stanford, California, USA.

**Keywords:** Immunology, Allergy, Cellular immune response, NK cells

## Abstract

Food allergies are a leading cause of anaphylaxis, and allergen-specific immune responses in both the innate and the adaptive immune system play key roles in its pathogenesis. We conducted a comprehensive phenotypic and functional investigation of immune cell responses from nonallergic (NA) and peanut allergic (PA) participants cultured with media alone or peanut protein and found, surprisingly, that NK cell activation was strongly associated with the immune response to allergen in PA participants. Peanut-responsive NK cells manifested a distinct expression pattern in PA participants compared with NA participants. Allergen-activated NK cells expressed both Th2 and immune regulatory cytokines, hinting at a potential functional role in mediating and regulating the Th2 allergic response. Depletion of CD3^+^ T cells attenuated the response of NK cells to peanut-allergen stimulation, suggesting that peanut-responsive NK cells are T cell dependent. We also showed that oral immune therapy was associated with decreased NK responses to peanut allergen stimulation in vitro. These results demonstrate that NK cells are associated with the food-allergic immune response, and the magnitude of this mobilized cell population suggests that they play a functional role in allergic immunity.

## Graphical abstract

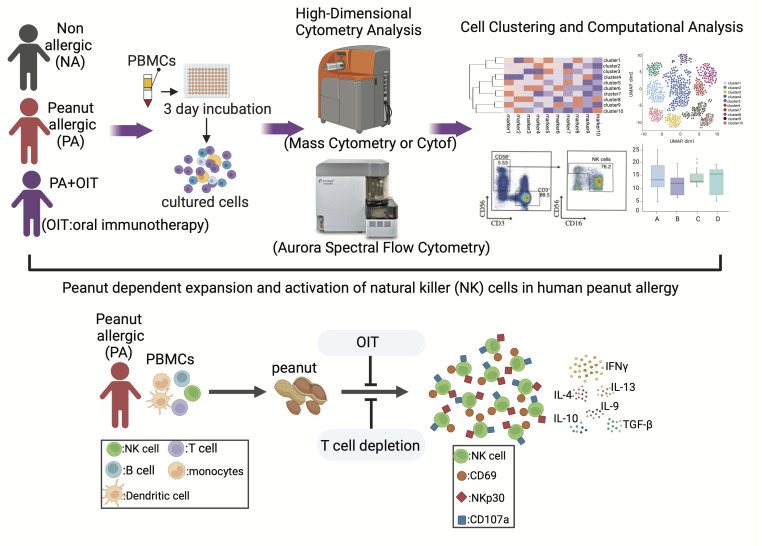

## Introduction

IgE-mediated food allergies may result in life-threatening anaphylaxis and affect up to 7.6% of children and 10.8% of adults in the United States (1, 2). The high prevalence and seriousness of this condition make it a major public health issue (1). Peanut allergies are the leading cause of allergy-related anaphylaxis and death in children and often persist into adulthood (3, 4). Treatment with oral immunotherapy (OIT) has recently been FDA approved for peanut allergy, and OIT in conjunction with either omalizumab or dupilumab has been employed in clinical trials (5–7).

The roles of T cells and the Th2-skewed immune response in IgE-mediated food allergies have been well established (8–11). Our group and others have shown that, after incubating PBMCs from individuals with PA with peanut protein, the frequencies of peanut-activated CD4^+^ T cells have increased (12–14), and peanut-reactive Th2 cells have decreased with OIT (15, 16).

Equally important to the pathogenesis of food allergy are cells of the innate immune system, including mast cells and basophils, which are well known effector cells of the allergic response, as well as group 2 innate lymphoid cells (ILC2s) and DCs (8, 17). Changes in monocytes have also been associated with food allergy. Newborns who eventually developed food allergy as well as 1-year old infants with established egg allergy both displayed a tendency toward a greater increase in inflammatory monocytes after nonspecific stimulation with LPS compared with nonallergic (NA) infants (18, 19). We reported that antigen-presenting cells, including monocyte-derived DCs, can reinforce the expansion of allergen-specific CD4^+^ T cells in PA individuals (20). These studies highlight the interaction and contribution of multiple cell types in the setting of food allergy. Human NK cells can regulate allergic inflammation through cytokine secretion and cell-cell contact-mediated mechanisms in allergic rhinitis, asthma, and atopic dermatitis (21). Recently, NK cells were shown to play an important regulatory role in atopic dermatitis pathogenesis through an NK cell–ILC2 inhibitory axis in mice and humans (22). However, little is known about the response of NK cells in the setting of food allergy.

Here, using high-dimensional, cytometry-based, single-cell analysis, we conducted a comprehensive survey of multiple immune cell populations for phenotypic and functional changes after food-allergen stimulation. We compared the response of PBMCs from NA and PA participants cultured in the presence of peanut protein or in media alone. Interestingly, we detected an increased frequency of NK cells in PA participants following stimulation of PBMCs with peanut protein. We confirmed this association between NK cells and peanut allergy by using 2 different statistical models (lasso logistic regression and random forest [RF] analyses) to identify activated NK cell populations as an important component that distinguishes the allergic response in PBMCs. Peanut-responsive NK cells from PA participants expressed Th2 cytokines as well as Th1 and immunomodulatory cytokines. Using Aurora spectral flow cytometry, we showed that peanut-responsive NK cells manifested a distinct expression pattern in PA compared with NA participants. We found that the responses of NK cells to peanut stimulation were reduced when CD3^+^ T cells were depleted from PBMCs before culture. Moreover, the responses of NK cells to peanut stimulation were reduced following treatment of PA participants with OIT.

## Results

### A survey of multiple immune cell subsets revealed an association between activated NK cell subsets and peanut allergen stimulation of PBMCs from PA participants.

To investigate the immune cell profile in response to food allergens, we performed comprehensive high-dimensional phenotypic and functional single-cell analysis using mass cytometry. PBMCs from NA and PA participants were cultured for 3 days in media alone or with peanut protein. Mass cytometry studies were performed with the hypothesis that peanut allergy–specific responses would be detected primarily in PBMCs from PA participants after culture with peanut protein.

The mass cytometry panel was designed to identify differences in the immune cell populations that comprise PBMCs, including CD4^+^ T cells, CD8^+^ T cells, NK cells, B cells, and myeloid cells. Further phenotyping of these populations was based on the expression of activation markers (CD154, CD69, and CD25), cell trafficking molecules (CLA, integrin β7, GPR15), cytokines, and other cell surface proteins such as the inhibitory receptor PD-1 and the senescence marker CD57 (Supplemental Table 1; supplemental material available online with this article; https://doi.org/10.1172/JCI157962DS1). Blood samples were taken from 22 PA participants who had avoided peanut exposure at baseline (with the exception of recent oral food challenge), and from 26 NA age- and sex-matched participants who had not avoided peanut exposure(Supplemental Table 2). Following in vitro culture of PBMCs and mass cytometry, we performed unsupervised Flow self-organizing maps–based (FlowSOM-based) clustering analyses, which identified 17 cell populations, or clusters, based on cell surface expression of lineage markers (Supplemental Figure 1A). The Uniform Manifold Approximation and Projection (UMAP) analysis was used to visualize high-dimensional single cell data (Supplemental Figure 1B) (23). The proportions of each cluster identified across each participant and experimental group is shown in Supplemental Figure 1C.

We next performed paired and unpaired comparisons between the 4 experimental groups — PA and NA, with and without peanut stimulation — using Wilcoxon signed rank and Wilcoxon rank sum tests. Among the 17 different clusters, we looked for cell population(s) demonstrating a significant increase in PA-derived PBMCs stimulated with peanut protein compared with PA-derived PBMCs cultured in media alone and NA-derived PBMCs. The only cell cluster fulfilling this criterion was the CD56^bright^CD16^–^ NK cell population (Supplemental Figure 1D). This result suggested that NK cells were associated with peanut allergy status.

**Figure 1:**
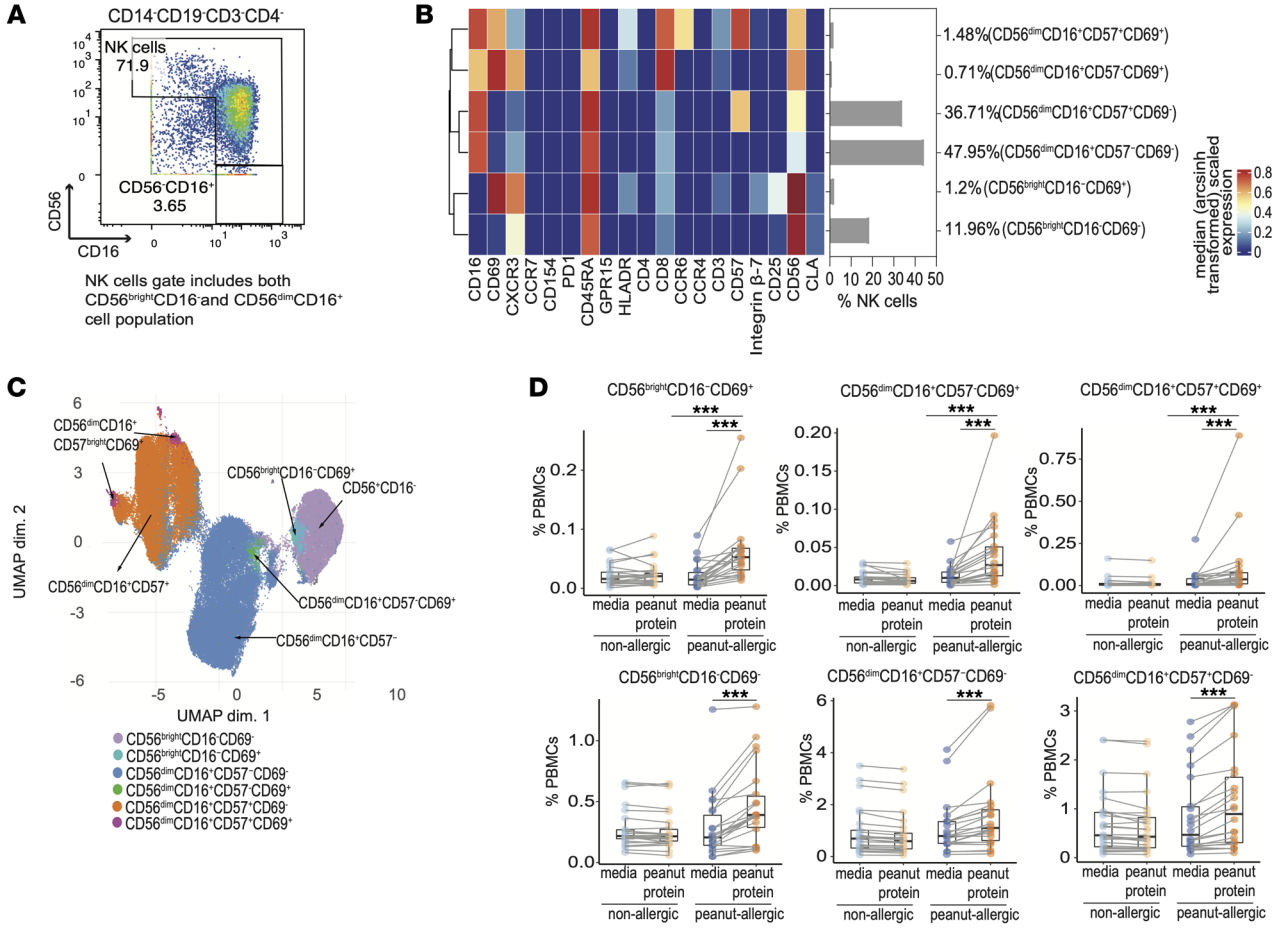
Peanut allergen stimulation is associated with activated NK cells in PBMCs from peanut-allergic participants, but not nonallergic participants. (**A**) Representative mass cytometry plots gated on CD14^–^CD19^–^CD3^–^CD4^–^ cells show the gating strategy for NK cells (NK cell gate includes both the CD56^dim^CD16^+^ and the CD56^bright^CD16^–^ NK subpopulations). (**B**) FlowSOM-based clustering analysis was performed on total pregated NK cells from PBMCs in nonallergic (NA) and peanut-allergic (PA) participants cultured with either media alone or peanut protein (*n* = 26 for NA PBMCs cultured with media alone or peanut protein; *n* = 22 for PA PBMCs cultured with media alone or peanut protein). Heatmap representing the median expression levels of cell surface markers in each cluster. The marker expression values were Arcsinh (inverse hyperbolic sine) transformed with a cofactor of 5. The bar graph shows each cluster as a percentage of total NK cells. (**C**) Uniform Manifold Approximation and Projection (UMAP) representation of 96,000 randomly selected cells (1,000 per sample; total 96 samples; *n* = 26 for NA PBMCs cultured with or without peanut protein, *n* = 22 for PA PBMCs cultured with or without peanut protein); clusters from the FlowSOM analysis are indicated by color. (**D**) The frequencies of 6 NK cell subsets as a percentage of total PBMCs from NA (*n* = 26) and PA (*n* = 22) participants with or without peanut stimulation. Each pair of points connected by a line represents 1 participant. Box plots indicate the interquartile range (IQR) and median; whiskers extend to the farthest data point within a maximum of 1.5 × IQR. Paired sample sets were analyzed using a 2-sided Wilcoxon signed rank test. Unpaired sample sets were analyzed using a 2-sided Wilcoxon rank sum test. *P* values were adjusted for multiple comparisons using the Bonferroni approach to control the FDR. FDR-adjusted *P* < 0.05 were considered significant. The asterisks indicate the FDR-adjusted *P* values, ***P < 0.001.

In the peripheral blood, 2 major subsets of NK cells exist: CD56^bright^CD16^–^ NK cells, which constitute approximately 10% of NK cells and preferentially secrete cytokines, and the dominant CD56^dim^CD16^+^ subset of NK cells that have high cytolytic capacity (24). We repeated the FlowSOM-based clustering analyses on manually gated NK cell subsets (Figure 1, A and B). The NK cell gate included both the CD56^dim^CD16^+^ and the CD56^bright^CD16^–^ NK subpopulations (Figure 1A). UMAP analysis was used to visualize the NK cell subsets at a high-dimensional single-cell level (Figure 1C). Six NK subsets were identified based on the expression of activation markers (CD69, CD25, and CD154), chemokine receptors (CCR7, CCR4, CCR6, and CXCR3), homing markers (GPR15, CLA, and integrin-β7), the inhibitory receptor PD-1, and CD57 (Figure 1, B and C). Of note, 3 of the 6 NK cell populations were characterized by the expression of CD69 (Figure 1, B and C).

We next performed paired and unpaired comparisons on the frequencies of these 6 NK subsets in total PBMCs for the 4 experimental groups using Wilcoxon signed rank and Wilcoxon rank sum tests. The frequencies of 3 NK cell populations (CD56^bright^CD16^–^CD69^+^, CD56^dim^CD16^+^CD57^–^CD69^+^, CD56^dim^CD16^+^CD57^+^CD69^+^) were increased in PA participants compared with NA participants in the peanut-stimulated PBMCs (Figure 1D). All 3 populations expressed CD69 while other NK populations did not, suggesting that these 3 NK populations may contribute to the response to food-antigen stimulation (Figure 1D).

### The magnitude of peanut dependent expansion was greater in CD69^+^ NK cells than in effector memory Th2 cells.

**Figure 2:**
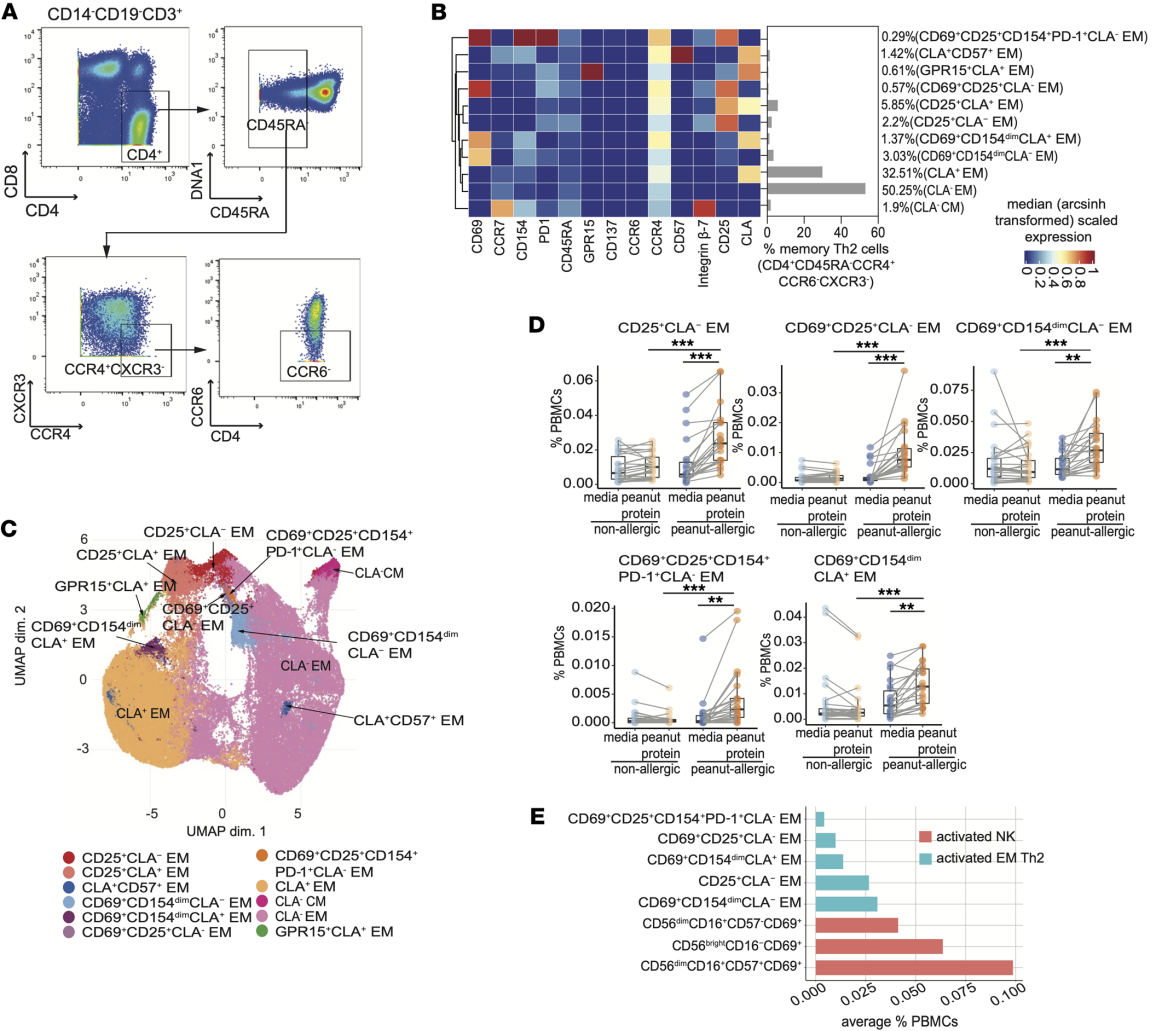
The magnitude of peanut-dependent expansion is greater in CD69^+^ NK cells than in effector memory Th2 cells. (**A**) Representative mass cytometry plots show the gating strategy for memory Th2 cells (CD14^–^CD19^–^CD3^+^CD4^+^CD45RA^–^CXCR3^–^CCR4^+^CCR6^–^). (**B**) FlowSOM-based clustering analysis was performed on gated memory Th2 cells from PBMCs from NA and PA participants cultured with media alone or peanut protein (*n* = 26 for NA PBMCs cultured with or without peanut protein, *n* = 22 for PA PBMCs cultured with or without peanut protein). Heatmap displays the median expression levels of cell-surface markers in each cluster. The marker expression values were arcsinh transformed with a cofactor of 5. The bar graph shows each cluster as percentage of memory Th2 cells. (**C**) UMAP representation of 96,000 randomly selected cells (1,000 per sample; total 96 samples, *n* = 26 for NA PBMCs cultured with or without peanut protein, *n* = 22 for PA PBMCs cultured with or without peanut protein); clusters from the FlowSOM analysis are indicated by color. (**D**) The frequencies of 5 memory Th2 cell subsets as a percentage of total PBMCs from NA (*n* = 26) and PA (*n* = 22) participants cultured with or without peanut stimulation. Each pair of points connected by a line represents 1 participant. Box plots indicate the IQR and median; whiskers extend to the farthest data point within a maximum of 1.5 × IQR. Paired sample sets were analyzed using a 2-sided Wilcoxon signed rank test. Unpaired sample sets were analyzed using a 2-sided Wilcoxon rank sum test. *P* values were adjusted for multiple comparisons using the Bonferroni approach to control the FDR. FDR-adjusted *P* < 0.05 were considered significant. The stars indicate the FDR-adjusted *P* values, ***P < 0.001 and **P < 0.01. (**E**) Average frequencies of activated NK cell subsets and activated EM CD4^+^ cell subsets as a percentage of total PBMCs from PA participants cultured with peanut protein.

We applied the same mass cytometric analyses to Th2 cells — which are commonly accepted to be integral to the pathophysiology of food allergy — in order to have a base with which to compare the increasing frequency of the magnitude and phenotype of the NK cell populations in this experimental system (13, 16, 25). The memory Th2 cell subset (26) was manually gated in the 4 experimental groups of PBMCs — PA and NA, with and without peanut protein (Figure 2A). FlowSOM-based unsupervised clustering analysis was applied using the same set of markers used for the NK cell analyses (Figure 2B), and UMAP analysis was used for visualization (Figure 2C). Eleven memory Th2 subpopulation clusters were identified (Figure 2, B and C). Among these, 6 cell subsets were effector memory (EM) Th2 cells that expressed different combinations of the activation markers CD69, CD25, and CD154 (Figure 2, B and C).

We applied Wilcoxon signed rank and Wilcoxon rank sum tests to perform paired and unpaired comparisons on the frequencies of the 11 memory Th2 subsets between the 4 experimental groups. In comparison with the NA with peanut protein group, the PA with peanut protein group showed significant increases in frequency in 5 out of the 6 activation marker-expressing EM Th2 subpopulations (Figure 2D and Supplemental Figure 2). These results are consistent with our previous work, which showed that the food allergen-specific immune response is composed of heterogenous, activated Th2 memory populations that express different activation marker combinations (CD69, CD25, CD154) (20, 27).

The frequencies of the 3 NK cell populations that expanded in culture with PBMCs and allergens (Figure 1D) were compared with those of the 5 allergen-responsive Th2 populations (Figure 2D) following peanut stimulation of PBMCs from PA participants. The 3 CD69^+^ NK cell populations had mean frequencies ranging from 0.04%–0.1% of total PBMCs, which were an order of magnitude higher than the mean frequencies of the 5 allergen-responsive EM Th2 populations, ranging from 0.004%–0.03% percent of total PBMCs (Figure 2E). Thus, the magnitude of the NK cell population activated in culture with allergens is greater than that of the allergen-specific Th2 population.

### Cytokine expression of NK cells was associated with peanut allergy status.

We analyzed cytokine expression of the CD69^+^ NK cell subsets, which expanded following peanut stimulation of PBMCs from PA participants. Because of the small number of activated Th2 EM cells, we compared this NK population with activated (CD69^+^) total EM CD4^+^ T cells.

**Figure 3:**
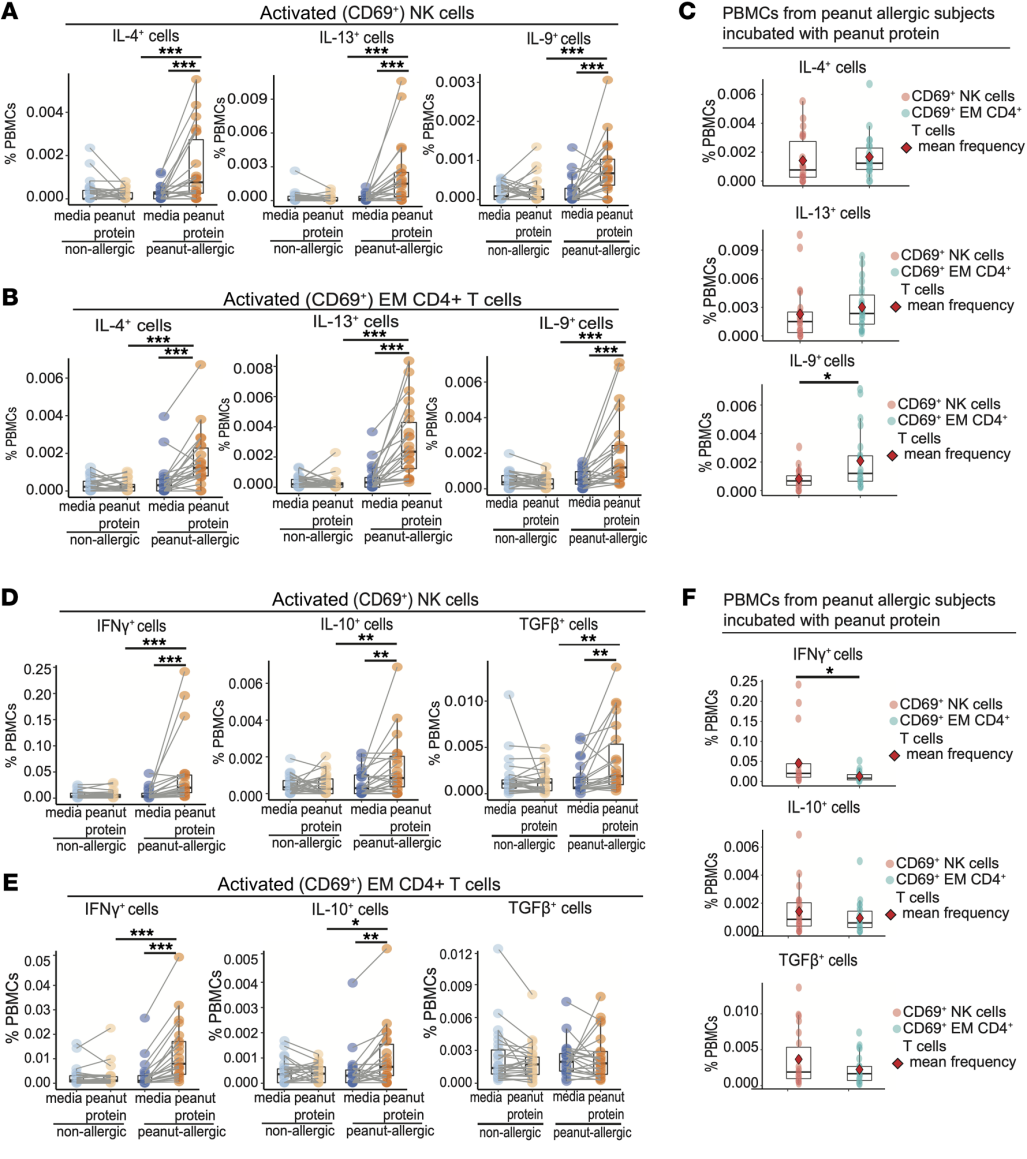
Cytokine-expression by NK cells is associated with peanut allergy status. (**A** and **B**) Allergen-specific Th2 cytokine expression (IL-4, IL-13, and IL-9) by activated NK cells (CD3^–^CD56^+^CD69^+^) (**A**) and activated EM CD4^+^ T cells (**B**). Frequencies are expressed as a percentage of total PBMCs from NA and PA participants cultured with or without peanut stimulation. Each pair of points connected by a line represents 1 participant. (**C**) Comparison of the frequency of Th2 cytokine expression between activated NK cells and activated EM CD4^+^ T cells. Each point represents 1 participant. The red rhombus represents the mean frequency. (**D** and **E**) Allergen-specific expression of the Th1 cytokine IFN-γ and immunomodulatory cytokines (IL-10 and TGF-β) by activated NK cells (**D**) versus activated EM CD4^+^ T cells (**E**). Frequencies are expressed as a percentage of total PBMCs from NA and PA participants with or without peanut stimulation. Each pair of points connected by a line represents 1 participant. (**F**) Comparison of the frequencies of Th1 and immunomodulatory cytokine expression by activated NK cells and activated EM CD4^+^ T cells. The red rhombus represents the mean frequency. Each point represents 1 participant. Box plots indicate the IQR and median; whiskers extend to the farthest data point within a maximum of 1.5 × IQR. Paired sample sets were analyzed using a 2-sided Wilcoxon signed rank test. Unpaired sample sets were analyzed using a 2-sided Wilcoxon rank sum test. ***P < 0.001, **P < 0.01, *P < 0.05.

The percentage of activated (CD69^+^) NK cells (Figure 3A) and activated (CD69^+^) EM CD4^+^ T cells (Figure 3B) that expressed the Type 2 cytokines IL-4, IL-13, and IL-9 increased significantly in the PA with peanut stimulation group compared with the other experimental groups. The average frequency of IL-4, IL-13, or IL-9-expressing activated NK cells was lower by 15% (*P* = 0.2), 24% (*P* = 0.1) and 61% (*P* = 0.02) respectively, than that of activated EM CD4^+^ T cells (Figure 3C). Thus, the number of Type 2 cytokine–expressing NK cells following peanut stimulation of PBMCs from PA participants is comparable to that of EM CD4^+^ T cells, and therefore is of a potential magnitude to affect allergic immunity.

Both NK and EM CD4^+^ T cells that were activated in culture with peanut allergen showed a significant increase in the expression of IFN-γ and IL-10 after peanut stimulation, while only NK cells showed a significant increase in TGF-β (Figure 3, D and E). The frequencies of activated NK cells that expressed the immunoregulatory cytokines IL-10 and TGF-β and the Type 1 cytokine IFN-γ were higher than those of activated EM CD4^+^ T cells. Specifically, the average proportion of activated NK cells that expressed IFN-γ, IL-10, or TGF-β increased by 72% (*P* = 0.01), 33% (*P* = 0.36), and 39% (*P* = 0.34), respectively, in comparison with the corresponding activated EM CD4^+^ T cells (Figure 3F). Thus, similar numbers of NK cells express immunomodulatory and Th1 cytokines in comparison with EM CD4^+^ T cells, suggesting that NK cells may have functional and regulatory effects on allergic immunity.

To compare the cytokine expression between activated (CD69^+^) NK cells and activated (CD69^+^) EM Th2 cells, we repeated the peanut stimulation with 5 NA and 7 PA participants (Supplemental Table 3) in which we aimed to achieve enough activated (CD69^+^) EM Th2 cells for gating the cytokine positive cells. Using Aurora spectral flow cytometry (Supplemental Table 4-panel 1 and panel 2), we acquired up to 1×10^6^ cells per sample. The frequency and absolute number of cytokine-expressing (IL-4, IFN-γ, IL-10, or TGF-β)CD69^+^ NK cells (Supplemental Figure 3, A and B) or CD69^+^ EM Th2 cells(Supplemental Figure 4, A and B) were increased significantly in PA with peanut stimulation group compared with the other experimental groups. The average frequency of IL-4–expressing CD69^+^NK cells was lower by 59% (*P* = 0.20) than that of CD69^+^EM Th2 cells, while the average frequency of IFN-γ, IL-10, or TGF-β–expressing CD69^+^ NK cells was higher by 834% (**P = 0.0012), 42% (*P* = 0.71), and 97% (*P* = 0.45), respectively, in comparison with the corresponding CD69^+^EM Th2 cells (Supplemental Figure 4C). Consistent with our results in cytometry by time of flight (CyTOF) experiments, similar numbers of CD69^+^ NK cells expressed IL-4, IL-10, and TGF-β, as did CD69^+^EM Th2 cells (Supplemental Figure 4C), whereas the frequency of IFN-γ–expressing CD69^+^ NK cells was significantly higher than that of IFN-γ-expressing CD69^+^ EM Th2 cells (Supplemental Figure 4C). To evaluate cytokine production per cell, we compared the median fluorescence intensity (MFI) of cytokines in cytokine-expressing activated NK cells and activated EM Th2 cells. We found that the IFN-γ MFI in IFN-γ–expressing CD69^+^ NK cells was significantly higher than that of IFN-γ–expressing CD69^+^ EM Th2 cells, while the MFI of IL-4, IL-10, and TGF-β in cytokine-expressing CD69^+^ NK cells was comparable to the MFI of those cytokines in CD69^+^ EM Th2 cells (Supplemental Figure 4D).

### NK cell subsets were an important component for the identification of the allergic immune response by Lasso logistic regression or RF analyses.

**Figure 4:**
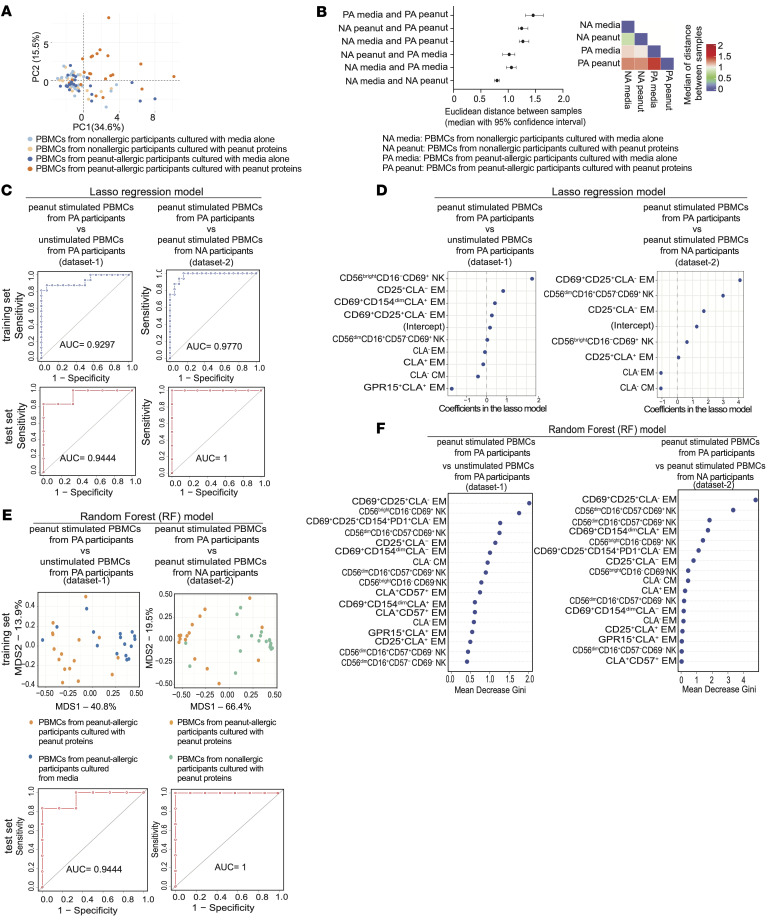
NK cell subsets are an important component for the identification of the allergic immune response by Lasso logistic regression or random forest analyses. (**A**) PCA of 17 cell subsets (6 NK cell subsets and 11 Th2 cell subsets) using data from 26 NA and 22 PA participants, cultured with or without peanut protein. The percentage variance explained by PC1 and PC2 are indicated. Each point represents 1 sample. (**B**) Euclidean distances computed based on 17 NK and Th2 cell subsets. The median values (with 95% CI) of the Euclidean distances between sample pairs are shown (left), and the median distances are displayed as a heat map (right). (**C**) Receiver operating characteristic (ROC) curves show the AUC, sensitivity and specificity of Lasso regression models for the training set (top) and test set (bottom). Left (data set 1) shows PBMCs from PA participants, peanut stimulated and unstimulated. Right (data set 2) shows peanut stimulated PBMCs from PA and NA participants. (**D**) Lasso regression model plots show the relative contribution (nonzero coefficient values) of different cell subsets in distinguishing peanut stimulated versus unstimulated PBMCs from PA participants (left), or peanut stimulated PBMCs from NA versus PA participants (right). The less contributive variables shrink to zero and are removed from the models. (**E**) Random forest (RF) models (using proximity scores from the training data set) were used to generate MDS plots that distinguish peanut stimulated versus unstimulated PBMCs from PA participants (top left), or peanut stimulated PBMCs from PA versus NA participants (top right). The RF models were used on the test data set to generate ROC curves showing the AUC, sensitivity and specificity. Bottom left shows peanut-stimulated versus unstimulated PBMCs from PA participants; bottom right shows peanut-stimulated PBMCs from PA versus NA participants. (**F**) Relative importance of different cell subsets used by the RF model to distinguish peanut-stimulated versus unstimulated PBMCs from PA participants (left), or peanut stimulated PBMCs from NA versus PA participants (right).

We employed an analytic approach to determine whether NK cell responses could be used to distinguish an allergic response to a food allergen. For our statistical analyses, we used the 17 cell subsets (6 NK cell subsets [Figure 1] and 11 memory Th2 cell subsets [Supplemental Figure 2]) which were identified through FlowSOM-based clustering. An unsupervised principal component analyses (PCA) of these subsets revealed that first and second principal components accounted for 50.1% of the variance in the data set and separated the majority of peanut-stimulated PA samples from the other groups (Figure 4A). A Euclidean-based distance analysis using the frequencies of these 17 cell subsets between the 4 groups also showed a greater separation of peanut-stimulated PBMCs from PA participants and the other groups (Figure 4B).

We attempted to identify which of the 17 cell subsets best distinguished peanut-stimulated PBMCs from unstimulated PBMCs from PA participants (data set 1); we additionally attempted to identify the subsets that best distinguished peanut-stimulated PBMCs from PA participants compared with those that identified peanut stimulated PBMCs from NA participants (data set 2). The mass cytometry results were split randomly into 2 data sets: 70% of the results were used as a training set, while the remaining 30% of the results were used to test model accuracy and predictive performance (test set). Two classification methods were used with the goal that they would mutually corroborate the important contributive variables for classification. The first method, penalized logistic regression analyses, allowed coefficients of the less-contributive variables to be shrunk to zero (28). The second model, RF analysis, allowed the importance of each classifying variable to be quantified (29).

The regularized (penalized) logistic regression model balances the smallest mean-squared error and smallest number of variables (cell subsets). Different types of regularized models were evaluated by explicitly controlling the fold of cross-validation for each observation. Results showed that the lasso (α = 1) models were better than elastic (α = 0.5) or ridge (α = 0) models for both data set 1 (Supplemental Figure 5A) and data set 2 (Supplemental Figure 5B). We then performed lasso logistic regression (α = 1) with 10-fold cross validation using the λ value that produced the most regularized model, such that the cross-validated error was within 1 standard error of the minimum (lambda.1se). These 2 lasso logistic regression models were built using the training data from the 17 cell subsets mentioned above to classify cell subsets from peanut-stimulated and unstimulated PBMCs from PA participants (data set 1) (Supplemental Figure 5C), or cell subsets from peanut-stimulated PBMCs from PA and NA participants (data set 2) (Supplemental Figure 5D). Based on the training data sets, these lasso models distinguished peanut-stimulated PA participants from unstimulated PA participants with an AUC of 0.9297 (Figure 4C, top left), or from peanut stimulated NA participants with an AUC of 0.9770 (Figure 4C, top right). When applied to the testing data sets, these models separated the same experimental groups with AUCs of 0.9444 (Figure 4C, bottom left) and 1 (Figure 4C, bottom right), respectively. In the final lasso models, the original 17 variables (cell subsets) were reduced to 9 variables (cell subsets) and 7 variables (cell subsets) for data sets 1 and 2, respectively (Figure 4D). The CD56^bright^CD16^–^CD69^+^ NK cell subset was the highest variable showing positive correlation with the classification of peanut-stimulated and unstimulated cells from PA participants (Figure 4D, left). The CD25^+^CD69^+^CLA^–^ EM Th2 cells were the first most-important variable, as might have been expected; however, CD56^dim^CD16^+^CD57^–^CD69^+^ NK cell subset was the second most-important variable, showing positive correlation with the classification of cell subsets from peanut-stimulated PBMCs from PA and NA participants (Figure 4D right).

RF models were constructed using training data from data sets 1 and 2 to classify the peanut-stimulated PBMCs from PA participants from unstimulated PBMCs from PA participants or from peanut-stimulated PBMCs from NA participants (Supplemental Figure 5, E and F). RF models were selected by tuning the number of variables randomly selected at each node splitting step to yield the smallest out-of-bag error (Supplemental Figure 5, E and F) (30–32). Using the training data sets, multidimensional scaling (MDS) plots based on proximity scores derived from the optimized RF models showed that peanut-stimulated PBMCs from PA participants can be separated from unstimulated PBMCs from PA participants (Figure 4E, top left) or from peanut-stimulated PBMCs from NA participants (Figure 4E, top right). Using the test data sets, these 2 RF models accurately distinguished peanut-stimulated from unstimulated PBMCs from PA participants (AUC of 0.9444) (Figure 4E, bottom left) or from peanut stimulated PBMCs from NA participants (AUC of 1) (Figure 4E, bottom right). The Gini measure of impurity is used to select the split with the lowest impurity at every node when constructing the RF model. The higher the value of mean decrease Gini score, the higher the importance to our model the variable would be (33). In Figure 4F, variable importance plots show the effect of each variable (cell subsets) on the accuracy of the RF models. The CD56^bright^CD16^−^CD69^+^ NK cell cluster was the second most-important variable for the classification of peanut-stimulated versus unstimulated PBMCs from PA participants (Figure, 4F left), with CD25^+^CD69^+^CLA^–^ EM Th2 cells being the first most-important variable. The CD56^dim^CD16^+^CD57^–^CD69^+^ NK cell subset was the second most-important variable for the classification of peanut-stimulated PBMCs from PA participants versus NA participants, with CD25^+^CD69^+^CLA^–^ EM Th2 cells being the first most-important variable (Figure 4F, right).

Importantly, the 2 different statistical models (lasso regression and RF) are in agreement that the same activated NK cell subsets, i.e., CD56^bright^CD16^–^CD69^+^ NK cells and CD56^dim^CD16^+^CD69^+^CD57^–^ NK cells, can distinguish responses of peanut stimulated PBMCs from PA participants from unstimulated PBMCs from PA participants or from peanut stimulated PBMCs from NA participants, respectively. The consistency of this result supports the association of specific NK cell populations with the immune response to food allergens (Figure 1).

### NK cells manifested a distinct expression pattern in PA compared with NA individuals.

**Figure 5:**
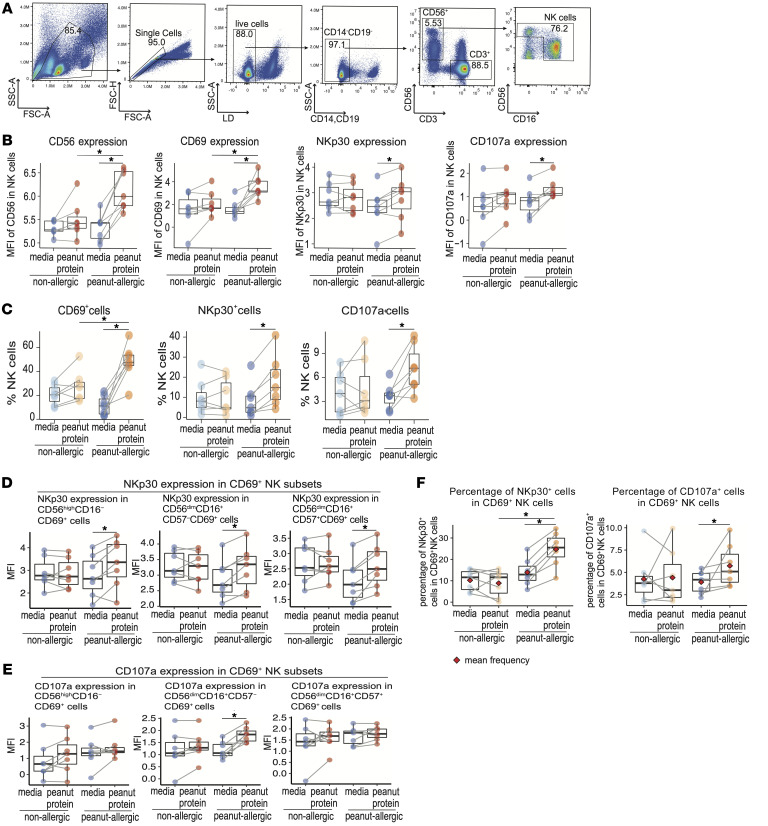
NK cells manifest a distinct expression pattern in PA compared with NA individuals. (**A**) Representative flow cytometry plots show the gating strategy for NK cells (**B**) Median fluorescence intensity (MFI) of CD56, CD69, CD107a and NKp30 on NK cells in NA (*n* = 7) and PA (*n* = 7) samples stimulated with or without peanut protein. The arcsinh transformation using standard cofactor 150 was applied for the expression value of each marker. (**C**) Frequencies of CD69^+^ NK cells, CD107a^+^ NK cells and NKp30^+^ NK cells among NK cell population cultured with or without peanut protein from NA (*n* = 7) versus PA participants (*n* = 7) (**D** and **E**) MFI of NKp30 (**D**) and CD107a (**E**) on CD69^+^ NK subsets cultured with or without peanut protein from NA (*n* = 7) versus PA participants (*n* = 7). (**F**) The NKp30^+^ cells or CD107a^+^ cells were gated on CD69^+^ NK cells. Frequencies of the NKp30^+^ cells and CD107a^+^ cells among the CD69^+^ NK cell population cultured with or without peanut protein from NA (*n* = 7) and PA participants (*n* = 7). The red rhombus represents the mean frequency. Box plots indicate the IQR and median; whiskers extend to the farthest data point within a maximum of 1.5 × IQR. Paired sample sets were analyzed using a 2-sided Wilcoxon signed rank test. Each pair of points connected by a line represents 1 participant. Paired sample sets were analyzed using a 2-sided Wilcoxon signed rank test. Unpaired sample sets were analyzed using a 2-sided Wilcoxon rank sum test. *P < 0.05.

We examined expression of the NK cell receptors NKp30, NKp46, NKG2C, and KIR [KIR2DL2/L3/KIR3DL1] (34), a functional marker of NK cell activity (CD107a) (35), along with the lytic activity marker (perforin), (36) simultaneously using Aurora spectral flow cytometry (Supplemental Table 4, panel 3) on PBMCs from 7 NA and 7 PA participants cultured in media alone or with peanut protein. We applied the arcsinh (inverse hyperbolic sine) transformation using standard cofactor 150 on the expression value of each marker per cell within the gated NK cells (Figure 5A), then calculated the MFI of each marker per sample. Consistent with the increased expression of CD56 and CD69 observed in NK cells using CyTOF (Supplemental Figure 6A), the expression levels of these 2 markers in NK cells were increased significantly in PBMCs from PA participants when cultured with peanut allergen compared with culture in media alone. The difference between PA participants’ PBMCs cultured with peanut protein and PBMCs from NA participants cultured with peanut protein was also significant (Figure 5B). Expression levels of NK receptor-NKp30 and the NK functional activity marker CD107a in total NK cells were increased following peanut stimulation in PA but not in NA participants compared with culture media alone (Figure 5B). However, the expression of CD16 and the NK receptors NKp46, NKG2C, KIR, and perforin in total NK cells remained unchanged after peanut protein stimulation (Supplemental Figure 6, B and C). We also quantified the frequency of CD69, NKp30, NKp46, NKG2C, KIR, CD107a, and perforin-expressing NK cells in the total NK cell population in PA and NA participants cultured with or without peanut protein. Consistent with the above results (Figure 5B), the frequencies of CD69^+^NK cells, NKp30^+^ NK cells, and CD107a^+^ NK cells in total NK cells were increased following peanut protein stimulation in PA participants (Supplemental Figure 7A and Figure 5C). The frequencies of NKp46, NKG2C, KIR, and perforin-expressing cells in total NK cells remained unchanged after peanut protein stimulation in PA participants (Supplemental Figure 7, A–C).

We applied FlowSOM-based clustering analysis on the gated NK cell population analyzed by flow cytometry (Figure 5A). Consistent with the NK subsets identified in CyTOF experiments (Figure 1, B and C), 6 NK subsets were identified based on the expression of CD56, CD16, CD57 and CD69 (Supplemental Figure 8, A and B). Of the 6 NK cell populations, 3 were characterized by the expression of CD69, and the frequency of these CD69-expressing NK cell subsets was significantly increased in PA-derived PBMCs after peanut stimulation compared with culture media alone (Supplemental Figure 8C). We further compared the expression levels of NKp30 and CD107a in CD69^+^ and CD69^–^ NK cell subsets. Following peanut stimulation, the expression of NKp30 was increased in all 3 CD69^+^ NK cell subsets (Figure 5D), while CD107a was increased only in the CD69-expressing CD56^dim^CD16^+^CD57^–^ NK cell subset (Figure 5E). The frequency of NKp30^+^ cells or CD107a^+^ cells in CD69^+^ NK cells was significantly increased following peanut stimulation in PA, but not in NA participants (Figure 5F). Following peanut stimulation, the average percentage of CD69^+^ NK cells expressing NKp30 and CD107 in PA participants was 24.74% and 5.73%, respectively (Figure 5F). The expression of NKp30 and CD107a remained unchanged in all 3 CD69^–^ NK cell subsets after peanut stimulation (Supplemental Figure 8, D and E). These results show that peanut allergen stimulation results in a distinct responsive expression pattern in NK cells in PA individuals compared with NA individuals.

### Peanut-responsiveNK cells expressed CD86.

**Figure 6:**
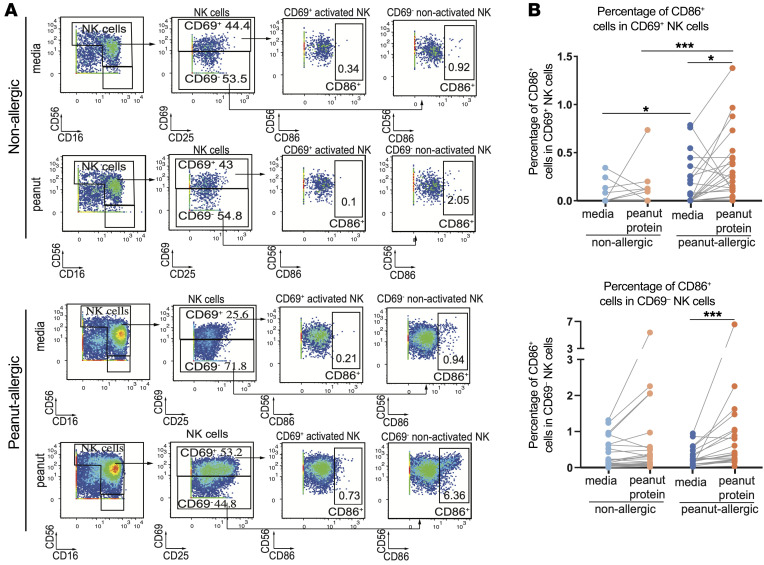
Peanut-responsiveNK cells express CD86. (**A**) Mass cytometry plots show the gating strategy for identifying CD86^+^ cells among activated and nonactivated NK cells from NA and PA participants cultured with or without peanut stimulation. The displayed values represent the expression frequency in the parent population. (**B**) Frequencies of the CD86^+^ cell population among activated (CD69^+^, top) and nonactivated NK cells (CD69^–^, bottom) cultured with or without peanut protein from NA and PA participants. Each pair of points connected by a line represents 1 participant. Paired sample sets were analyzed using a 2-sided Wilcoxon signed rank test. Unpaired sample sets were analyzed using a 2-sided Wilcoxon rank sum test. *P < 0.05, ***P < 0.001.

We sought to identify potential modes of interaction between NK cells and the adaptive immune system. We quantified the frequency of CD86^+^ cells in activated NK cells (CD69^+^ NK cells) and nonactivated NK cells (CD69^–^ NK cells) from PA and NA participants cultured with or without peanut protein (Figure 6, A and B). We found that the frequency of CD86^+^ cells both in activated NK cells (CD69^+^) and in nonactivated NK cells (CD69^–^ NK cells) was significantly increased following peanut stimulation compared with culture media alone in PA, but not in NA participants. The percentage of CD86^+^ activated NK cells was significantly increased after peanut stimulation in PA compared with NA participants (*P* < 0.001). Increased frequencies of both CD86-expressing NK cells (Supplemental Figure 9A) and B cells (Supplemental Figure 9B) were observed in PA participants following peanut stimulation compared with culture media alone and compared with NA participants cultured with peanut protein (Supplemental Figure 9D). In contrast, peanut stimulation induced an increased frequency of CD86-expressing myeloid cells in both NA and PA participants, suggesting that the changes of CD86^+^ myeloid cells are not influenced by allergic status (Supplemental Figure 9, C and D). Thus, CD86 expression represents a potential link to the adaptive immune system that could be involved in the NK response to peanut observed in PA participants.

### Peanut-responsive NK cells were T cell dependent.

**Figure 7:**
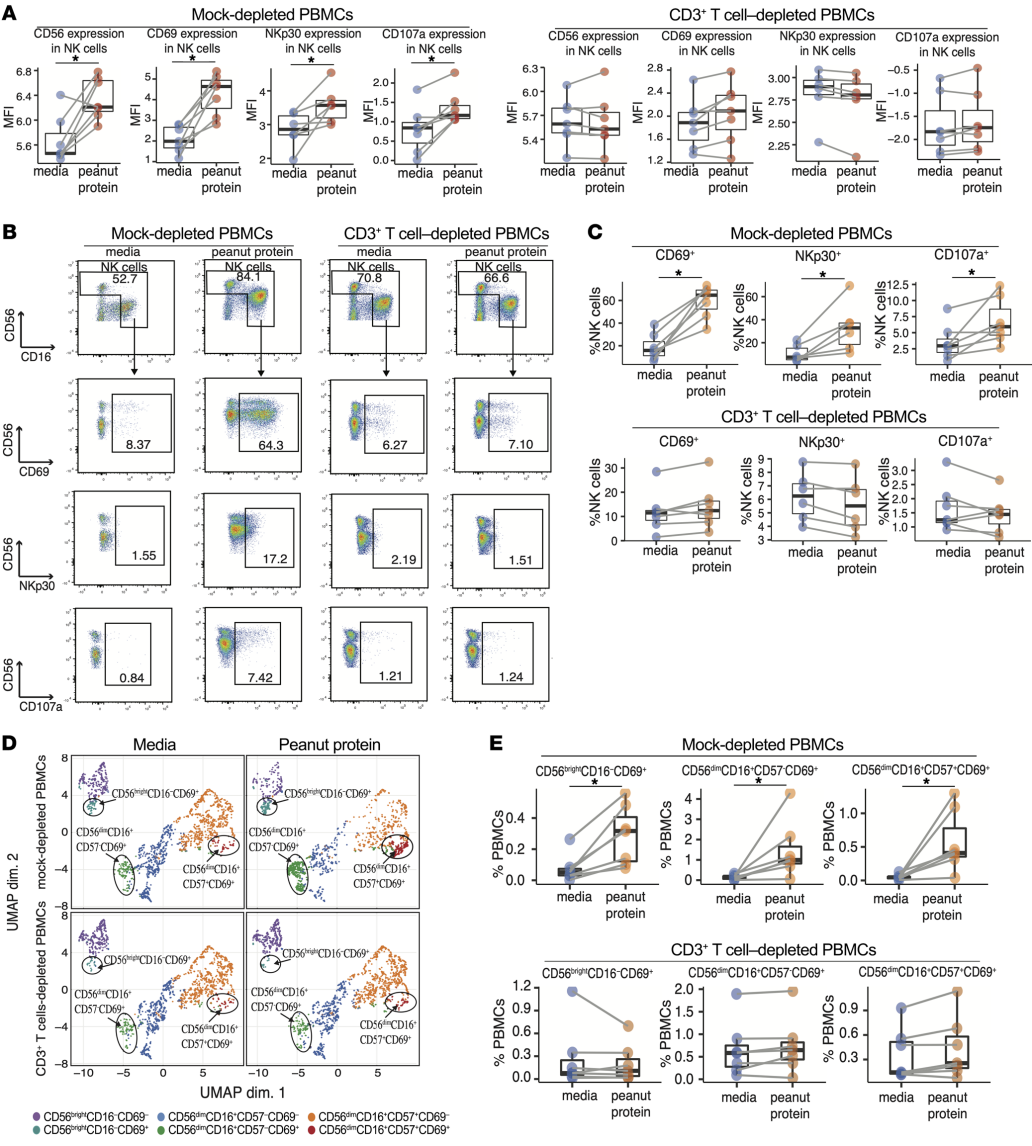
Depletion of CD3^+^ T cells attenuates NK cell activation. (**A**) MFI of CD56, CD69, CD107a and NKp30 on NK cells in mock-depleted PBMCs (right) and CD3^+^ T cells depleted PBMCs (left) stimulated with or without peanut protein from PA (*n* = 7) participants. (**B** and **C**) Representative flow cytometry plots (**B**) and boxplot overlaid with dot plots (**C**) show the gating strategy and frequency of CD69^+^ NK cells, CD107a^+^ NK cells and NKp30^+^ NK cells among NK cell population in mock-depleted PBMCs and CD3^+^ T cell–depleted PBMCs cultured with or without peanut protein. (**D**) UMAP representation of 7,000 randomly selected cells; 250 cells per sample, 28 total samples in 4 experimental groups including mock-depleted PBMCs, CD3^+^ T cell–depleted PBMCs, each with and without peanut protein stimulation; clusters from the FlowSOM analysis are indicated by color. (**E**) The frequency of CD69^+^ NK subsets in mock-depleted PBMCs versus CD3^+^ T cell–depleted PBMCs. Box plots indicate the IQR and median; whiskers extend to the farthest data point within a maximum of 1.5 × IQR. Each pair of points connected by a line represents 1 participant. Paired sample sets were analyzed using a 2-sided Wilcoxon signed rank test. Paired sample sets were analyzed using a 2-sided Wilcoxon signed rank test. *P < 0.05.

In the adaptive immune system, Th2 cells, as well as other allergen-responsive T cell types, such as Tregs (15, 37), follicular helper T (Tfh) cells (38, 39), and CD8^+^ T cells (40, 41) contribute to the allergic immune response. To interrogate the role of T cells on the changes in the peanut-responsive NK cells, we depleted CD3^+^ T cells from the PBMCs (Supplemental Figure 10A) of 7 PA participants (Supplemental Table 3) before incubation with or without peanut protein for 3 days. Depletion of CD3^+^ T cells inhibited the peanut-induced increases in the expression levels of CD56, CD69, NKp30, and CD107a in NK cells (Figure 7A) and the increased frequencies of CD69^+^, NKp30^+^, or CD107a^+^ NK cells that were observed in the control, mock-depleted PBMCs after peanut stimulation (Figure 7, B and C).

We repeated the FlowSOM-based clustering analysis on the gated NK cell population from PA-derived, mock-depleted PBMCs and CD3^+^ T cell–depleted PBMCs in the flow cytometry experiment. Again, 6 NK subsets were identified based on the expression of CD56, CD16, CD57, and CD69 (Supplemental Figure 10, B and C). Consistent with the NK subsets identified from FlowSOM clustering analysis in Figure 1, we observed increased CD69-expressing NK subsets in response to peanut stimulation in mock-depleted PBMCs (Supplemental Figure 10D and Figure 7, D and E). However, these CD69^+^ NK subsets in CD3^+^ T cell–depleted PBMCs did not show an increased frequency following peanut stimulation (Figure 7, D and E).

**Figure 8:**
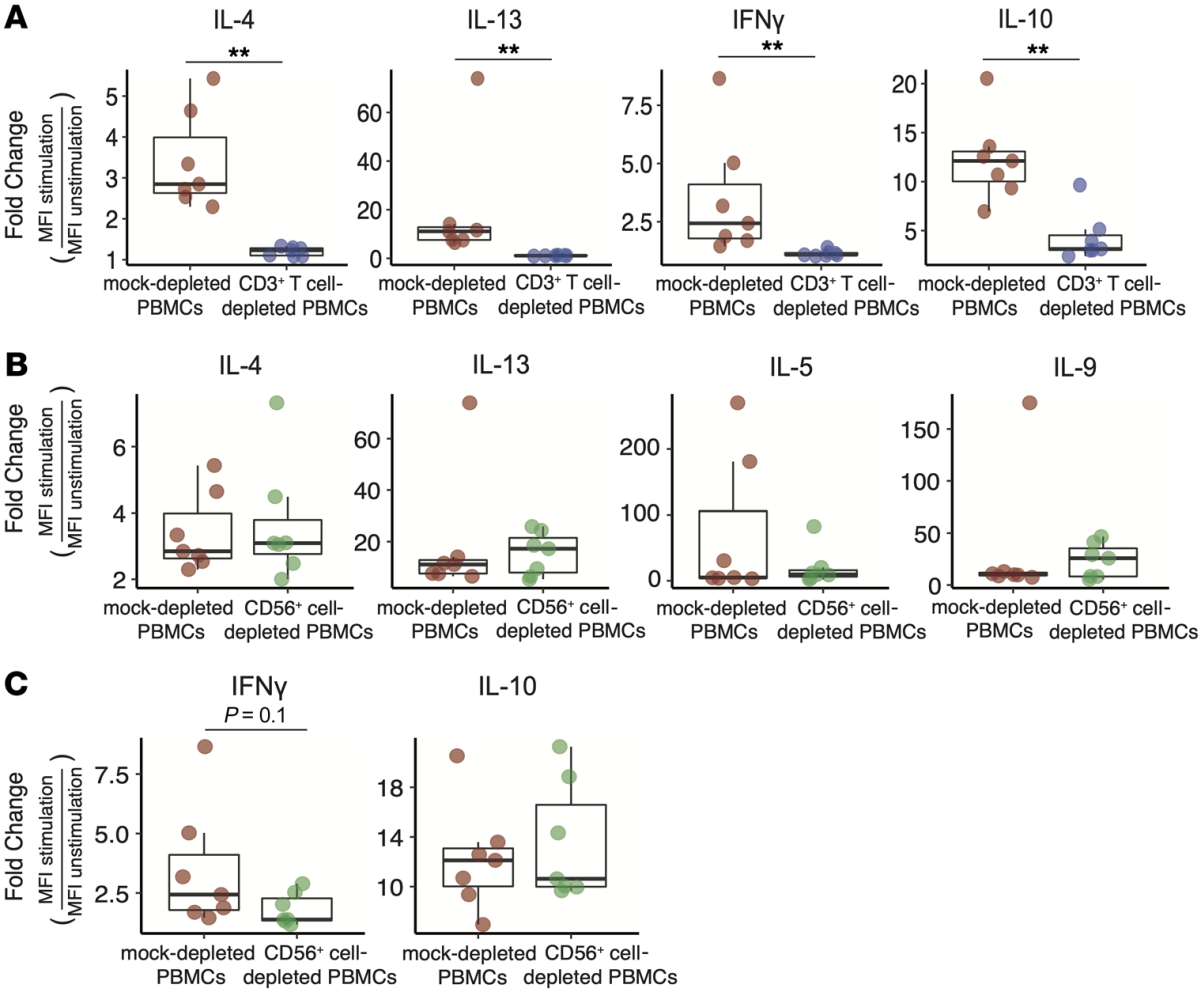
Cytokine secretion in response to stimulation with peanut allergen was altered in CD3^+^ T cell–depleted or CD56^+^ cell-depleted PBMCs compared with mock-depleted PBMCs. (**A**) The fold change of peanut-induced cytokines (IL-4, IL-13, IFN-γ, and IL-10) in CD3^+^ T cell–depleted PBMCs and mock-depleted PBMCs. (**B** and **C**) The fold change of peanut-induced cytokines (IL-4, IL-13, IL-9, IL-5, IFN-γ, and IL-10) in PBMCs depleted of CD56^+^ cells versus mock-depleted PBMCs. The fold change was calculated as the expression levels of these cytokines measured by Luminex assay in the culture with peanut protein (stimulated group) divided by that in the culture with media (unstimulated group). Box plots indicate the IQR and median; whiskers extend to the farthest data point within a maximum of 1.5 × IQR. Unpaired sample sets were analyzed using a 2-sided Wilcoxon rank sum test. **P < 0.01.

We measured cytokine production in CD3^+^ T cell–depleted PBMCs (Supplemental Figure 10A), CD56^+^ cell–depleted PBMCs (Supplemental Figure 11A), and mock-depleted PBMCs from PA participants cultured with or without peanut protein using a Luminex-based assay. Consistent with the above results (Supplemental Figures 3 and 4), peanut stimulation induced the increased expression of these cytokines in mock-depleted PBMCs (Supplemental Figure 11B, top). We found that the secretion of cytokines IL-9, IL-5, and TGF-β from CD3^+^ T cell–depleted PBMCs (Supplemental Figure 11B, middle), and the secretion of TGF-β from CD56^+^ cell–depleted cells were no longer induced by peanut stimulation (Supplemental Figure 11B, bottom). To further compare cytokine expression between CD3^+^ T cell–depleted or CD56^+^ cell–depleted PBMCs and mock-depleted PBMCs, the fold change was calculated as the expression levels of these cytokines in the culture with peanut protein (stimulated group) divided by that in the culture with media (unstimulated group). The results showed that the secretion (fold change) of IL-4, IL-13, IFN-γ and IL-10 was significantly lower in CD3^+^ T cell–depleted PBMCs than that of mock-depleted PBMCs following peanut stimulation (Figure 8A), although detectable levels of these cytokines were observed (Supplemental Figure 11B, middle). In CD56^+^ cell-depleted PBMCs, the secretion (fold change) of cytokines (IL-4, IL-13, IL-9, IL-5, IFN-γ, and IL-10) induced by peanut stimulation was comparable to that of mock-depleted PBMCs (Figure 8B and Supplemental Figure 11B, bottom), whereas IFN-γ secretion (fold change) showed a decreasing trend compared with that of mock-depleted PBMCs (Figure 8C). These results suggested that depletion of CD3^+^ T cells suppressed the secretion of type 2 cytokines (IL-4, IL-13, IL-9, and IL-5), immunoregulatory cytokines (IL-10 and TGF-β), and the type 1 cytokine IFN-γ. Depletion of CD56^+^ cells was associated with the reduced secretion of TGF-β and IFN-γ after peanut stimulation, but did not influence the production of IL-4, IL-13, IL-9, IL-5, and IL-10.

### OIT was associated with diminished activation of peanut-responsive NK cells from PA participants cultured with peanut protein.

**Figure 9:**
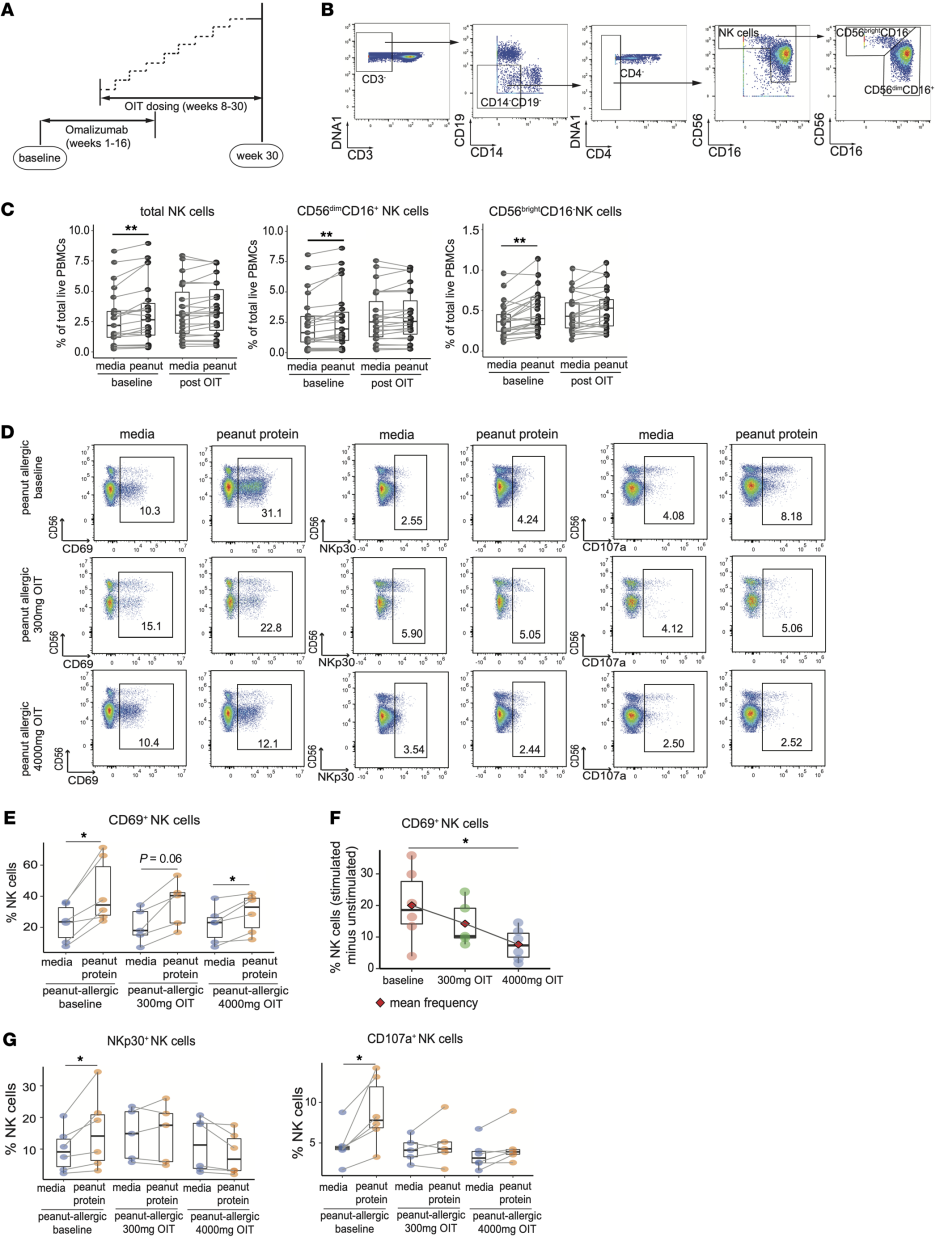
OIT is associated with diminished activation of NK cells from PA participants cultured with peanut protein. (**A**) Schematic of the timeline of the clinical study design, showing omalizumab injections from weeks 1–16 and OIT from weeks 8–30. Blood samples were drawn at baseline and week 30. (**B**) Mass cytometric gating strategy for NK cell subsets. (**C**) Total NK cells (left), CD56^dim^CD16^+^ NK cells (middle) and CD56^bright^CD16^dim/–^ NK cells (right) as a percentage of total PBMCs cultured with or without peanut protein at baseline and week 30 after OIT from 23 PA participants. (**D**) Representative flow cytometry plots gated on NK cells showing the effect of OIT on CD69^+^NK, NKp30^+^NK, and CD107a^+^NK for 1 PA participant. (**E**) Percentage of CD69^+^NK in NK cells shown for 6 PA participants before and during OIT. (**F**) The degree of increase in the CD69^+^ NK cells in response to peanut stimulation was calculated by subtracting the percentage of this population in the unstimulated sample from that in the corresponding stimulated sample. The degree of peanut-induced increase in CD69^+^ NK cells was examined before and during OIT for 6 PA participants. (**G**) Percentage of NKp30^+^NK (left) and CD107a^+^NK (right) in NK cells shown for 6 PA participants before and during OIT. Since the PBMC sample from 1 of the 6 PA participants at the 300 mg peanut OIT was not available, we only have 5 readouts at this timepoint. Box plots indicate the IQR and median; whiskers extend to the farthest data point within a maximum of 1.5 × IQR. Paired sample sets were analyzed using a 2-sided Wilcoxon signed rank test. Each pair of points connected by a line represents 1 participant. Unpaired sample sets were analyzed using a 2-sided Wilcoxon rank sum test. The stars indicate the P values, **P < 0.01.

OIT desensitizes individuals to food allergens, and its effects include a decreased Th2 response (15, 16, 42). To determine whether OIT has a similar effect on NK cells, we analyzed PBMCs from 23 PA participants before and after omalizumab-facilitated OIT to a maintenance dose of 1,000 mg peanut protein (Supplemental Table 5). Specifically, participants received omalizumab from week 0 to week 16 and OIT from week 8 to week 30 (Figure 9A).

As expected, following peanut stimulation, PBMCs from PA participants at baseline showed increased frequencies of total NK cells and the CD56^bright^CD16^–^ NK cell and CD56^dim^CD16^+^ NK cell subsets compared with culture in media alone (Figure 9, B and C). Following OIT, peanut stimulation of PBMCs from PA participants no longer induced a significant increase in NK cell subsets, suggesting that the allergen-desensitization procedure inhibited the NK response (Figure 9, B and C).

To rule out the influence of omalizumab on changes in NK cell frequencies, we performed the peanut protein–stimulation experiments on PBMCs from 6 PA children who received peanut OIT without omalizumab (Supplemental Table 6) (5). PBMCs from the 6 PA children before peanut OIT treatment, at 300 mg of peanut protein during OIT updosing, and at a maintenance dose of 4,000 mg peanut protein were incubated with or without peanut and analyzed by Aurora spectral flow cytometry (Supplemental Table 4-panel 3). Peanut stimulation induced a significant increase in the frequencies of total NK cells and CD56^bright^CD16^–^ NK subset in total PBMCs compared with the culture media alone at baseline, whereas the peanut-induced increase in the frequency of NK cells was inhibited in participants treated with either 300 mg peanut OIT or 4,000 mg peanut OIT (Supplemental Figure 12, A and B), which is consistent with the results from 23 PA participants who received omalizumab-facilitated OIT in the CyTOF experiment. Consistent with the above results (Supplemental Figure 7), the frequencies of NKp46, NKG2C or KIR-expressing NK cells were unchanged after peanut stimulation in PA participants at baseline as well as after peanut OIT (Supplemental Figure 12C). Peanut stimulation induced an increased frequency of CD69^+^ NK cells at baseline (Figure 9, D and E), and the degree of this increase in CD69^+^ NK cells was significantly lower at the maintenance dose of 4000mg when compared with the baseline (Figure 9F). A trend toward a reduction in the degree of this increase at the 300 mg updosing phase of OIT was observed (Figure 9F). In addition, increased frequencies of CD107a^+^ NK cells or NKp30^+^ NK cells cultured with peanut protein at baseline were no longer observed following treatment with either 300 mg peanut OIT or 4,000 mg peanut OIT (Figure 9G). These findings suggest that treatment of peanut allergy with OIT is associated with a decrease in NK responses to peanut allergen stimulation.

## Discussion

In this study we performed a comprehensive phenotypic and functional investigation of peanut-responsive NK cells in established IgE-mediated peanut allergy. Our results showed that CD69^+^, activated NK cells were increased after peanut stimulation in PA participants compared with NA participants (Figure 1) and that the magnitude of this increase was greater than that of peanut-responsive, activated EM Th2 cells (Figure 2E). The link between food allergy and NK cells is further supported by analyses using 2 different statistical methods (lasso logistic regression and RF analyses). Both statistical models identified the same activated NK cell subpopulations as the most important variables for distinguishing the PA with peanut versus PA with media groups (CD56^bright^CD16^–^CD69^+^ NK population) and the PA with peanut versus NA with peanut groups (CD56^dim^CD16^+^CD57^–^CD69^+^ NK population). We show that the peanut-responsive NK cells in PA participants are T cell–dependent. Treatment of PA children with peanut OIT suppressed the peanut-induced increase in the frequency of NK cells as well as the peanut-induced increase in CD69^+^ NK cells, CD107a^+^ NK cells, and NKp30^+^ NK cells observed at baseline, which suggests that the allergen-desensitization procedure inhibited the responses of NK cells to allergen stimulation.

Although best known for their role in infection (43) and ability to kill tumor cells (44), studies have shown that NK cells can participate in allergy-associated Th2 immunity (45, 46). In atopic dermatitis, NK cells play a critical immunoregulatory role and can act as a potential therapeutic target by enhancing immune pathways such as IL-15 (22). Finally, in an ovalbumin-induced murine model of allergic airway disease, NK-deficient mice had decreased airway inflammation and eosinophilia, decreased secretion of IL-4, IL-5, and IL-13, as well as diminished OVA-specific antibody production (47). A retrospective analysis of 121 children who were allergic to multiple foods reported a pattern of immune deviation consisting of a relatively decreased percentage of NK and CD8 cells and increased percentages of CD4 and CD19 cells (48). Neeland et al. showed that, following nonspecific stimulation of PBMCs with PMA/ionomycin, PA 1-year-old infants exhibited increased expression of TNFα in NK cells (12). Although these findings suggest a role for NK cells in allergic disease, these studies did not investigate NK cell responses to food allergens in food-allergic individuals.

The proportion of activated NK cells expressing Th2 cytokines is comparable to that of activated EM CD4^+^ T cells or activated EM Th2 cells, consistent with the possibility that NK cells are activated by culture of PBMCs with allergens in sufficient numbers to affect the immune response (Figure 3, A–C, and Supplemental Figures 3 and 4). Expression of the cytokines IL-4, IL-13, and IL-9 by allergen-activated NK cells suggests that this population may reinforce the Th2 allergic response to food allergens, which is consistent with previous studies supporting a role for NK cells in potentiating the allergic phenotype in other atopic diseases (21, 45).

Interestingly, we observed that a similar proportion of activated NK cells expressed IL-10 and TGF-β compared with activated EM CD4^+^ T cells or activated EM Th2 cells. The frequency of IFN-γ–expressing CD69^+^ NK cells was significantly higher than that of IFN-γ–expressing CD69^+^ EM CD4^+^ T cells or IFN-γ–expressing CD69^+^ EM Th2 cells (*P* = 0.0012). Moreover, the IFN-γ MFI in IFN-γ–expressing CD69^+^ NK cells was significantly higher than that of IFN-γ–expressing CD69^+^ EM Th2 cells (*P* = 0.0019). IFN-γ could be expected to skew the immune response from Th2 to Th1 and so might dampen food allergic responses (10, 11). Similarly, IL-10 and TGF-β are also thought to dampen immune responses (49). Thus, NK cells could conceivably exercise a regulatory role over Th2 cells and help modulate allergic immunity (50).

Peanut-responsive NK cells presented a distinct expression pattern in PA participants compared with NA participants; they show an increased expression level of CD56, CD69, the natural cytotoxicity receptor NKp30 (51), and CD107a, a marker of NK cell functional activity (35), while expression levels of NKp46, NKG2C, KIR (KIR2DL2/L3/KIR3DL1), and perforin were unchanged. CD69 and CD56 have been used as activation markers in PMA/ionomycin stimulated NK cells (52) and IL-12 activated NK cells (53). In our study, peanut-responsiveNK cells were characterized by the increased expression of CD56, CD69, NKp30 and CD107a in PA participants. Interestingly, NKp30 expression was increased in all 3 CD69^+^ NK subsets (CD69^+^CD56^high^CD16^–^, CD69^+^CD56^dim^CD16^+^CD57^–^ and CD69^+^CD56^dim^CD16^+^CD57^+^) (Figure 5D) but not in CD69^–^ NK cells, while CD107a expression was increased in only CD69^+^CD56^dim^CD16^+^CD57^–^ cells. These results suggest that, following peanut stimulation, NKp30-expressing, activated NK cells are associated with both cytokine secretion and cytotoxicity, while CD107a-expressing, activated NK cells may be related preferentially to cytotoxicity.

One hypothesis for the activation of NK cells upon culture of PA patients’ PBMCs with allergens is that the response of allergen-specific T cells activates and/or promotes survival of a subpopulation of NK cells. Depletion of CD3^+^ T cells from PBMCs of PA participants before incubation with peanut protein inhibited the peanut-induced NK expansion and cytokine response observed in mock-depleted PBMCs. These results suggest that the response of NK cells following peanut stimulation is T cell dependent and that activation of NK cells is a secondary downstream response, which is consistent with previous studies in which NK cell activation was regulated by effector CD4^+^ T cells (54, 55). Interestingly, CD86 was preferentially upregulated by NK cells from allergic individuals after incubation with peanut protein. Expression of CD86, the ligand for the classical TCR costimulatory receptor CD28, has been shown to increase in activated NK cells (56). Binding of CD86 on NK cells has been shown to induce activation (57, 58), suggesting that CD28/CD86 binding might contribute to NK activation by allergen-specific T cells.

Invariant NK T (*i*NKT) cells are CD3^+^ and express an invariant aβ T cell receptor (TCR) that recognizes lipid antigens. *i*NKT cells also express CD56 and other molecules in common with NK cells. Two recent studies introduced a connection between Th2 responses and *i*NKT in allergic disease (59, 60). Although a role of *i*NKT cells in food allergy has been postulated given their recognition of lipid antigens, Chu et al. reported that IL-4–dependent PA Th2 responses were completely intact in NKT-deficient mice and were dependent on OX40L expression by DCs (59). Consistent with this, our results showed that depletion of CD56^+^ cells had no effect on the peanut-induced increase in the production of Th2 cytokines (IL-4, IL-5, IL-9, and IL-13) and the immunoregulatory cytokine IL-10, suggesting that T cells are the major source of these cytokines in response to peanut stimulation in PA individuals. Our results also showed that removing CD56^+^ cells was associated with reduced production of TGF-β and IFN-γ by peanut stimulation, suggesting that CD56^+^ cells may play a regulatory role in the modulation of Th2-skewed immune response in peanut allergy by secretion of TGF-β and IFN-γ.

Overall, our results demonstrate that NK cell activation is strongly associated with the immune response to allergen in peanut allergy. NK cells activated in culture with peanut protein express Th2 cytokines as well as immunomodulatory cytokines, suggesting a potential functional role for this population in mediating and regulating Th2 allergic response. Peanut-responsive NK cells present a distinct expression pattern in PA compared with NA participants. The responses of NK cells to peanut allergen are T cell–dependent, and the frequency of CD86-expressing NK cells from allergic individuals is upregulated after incubation with peanut protein, suggesting a potential mechanism for NK cell activation. Treatment of peanut allergy with OIT blunts the peanut-induced responses of NK cells, further highlighting the association between NK cells and food allergy.

## Methods

### Study participants.

PA and NA pediatric participants were recruited at the Sean N. Parker Center for Allergy and Asthma Research at Stanford University. Patient demographics, food allergy history, atopic history, and peanut-specific IgE are summarized in Supplemental Tables 2, 3, 5, and 6. Peanut allergy was confirmed by a double-blind, placebo-controlled food challenge (DBPCFC). PA participants with omalizumab-facilitated OIT or peanut OIT alone were taken from a random sampling of participants in a previously published, phase II, randomized controlled study (5, 6).

### Cell preparation.

For the PA participants shown in Supplemental Tables 2 and 3, blood samples were taken from the allergic participants avoiding peanut at baseline (with the exception of recent oral food challenge). For the PA participants shown in Supplemental Table 5, blood samples were taken from the allergic participants before peanut OIT treatment (at baseline) and after omalizumab-facilitated OIT at week 30. For the PA participants shown in Supplemental Table 6, blood samples were taken from the allergic participants before peanut OIT treatment (at baseline), during OIT updosing phase at 300 mg of peanut protein, and at a maintenance dose of 4000 mg peanut protein. Since the PBMC sample from 1 of 6 PA participants at 300 mg peanut OIT was not available, we have 5 readouts for this time point. PBMCs were isolated from blood samples by density gradient centrifugation over Ficoll-Paque, cryopreserved in 10% DMSO in fetal calf serum and stored in liquid nitrogen.

### Cell isolation using magnetic sorting.

CD3^+^ T cell– or CD56^+^ cell–depleted PBMCs were obtained by using CD3 or CD56 magnetic microbeads (Miltenyi Biotec) to remove CD3^+^ T cells or CD56^+^ cells from PBMCs, respectively. Following depletion, 99.97% of CD3^+^ T cells (Supplemental Figure 10A) and 99.51% of CD56^+^CD3^–^ cells (Supplemental Figure 11A) were removed from whole PBMCs. The mock-depleted PBMCs were from the same PA participants and were treated in parallel but without the addition of CD3 or CD56 microbeads.

### In vitro stimulation.

Peanut protein solution added to cell culture was prepared from the peanut flour used for DBPCFC in clinic, dissolved in PBS and sterilized by filtration as previously described (20). PBMCs from NA and PA participants were thawed. The whole PBMCs, mock-depleted PBMCs, CD3^+^ T cell–depleted PBMCs, or CD56^+^ cell-depleted PBMCs were plated in 96 well, round-bottomed plates with 4–5×10^5^ cells in 200μl of complete RPMI medium (RPMI 1640 medium, 5% human serum (Sigma-Aldrich), 1% penicillin/streptomycin (ThermoFisher Scientific) per well. Cells were plated in equal number per each culture condition for the same sample, as determined by the available quantity of the cryopreserved PBMCs from different samples. After overnight resting, the PBMCs were cultured in the presence or absence of peanut protein for 3 days. Peanut protein was added to the culture at a final concentration of 100 μg/mL. After 3 days of culture, brefeldin A (BFA, 1 μg/mL; Biolegend) was included during the last 4 hours of the cell culture.

### Mass cytometry.

Antibodies purchased from Fluidigm or conjugated with the lanthanides using MAXPAR antibody labeling kit (Fluidigm) following the manufacturer’s protocol are listed in Supplemental Table 1. Antibodies were titrated prior to use. After 3 days of culture, cell harvesting, staining, data acquisition, and preprocessing were performed as previously described (20). The intact live cells were gated to exclude normalization beads, dead cells, and doublets using Flowjo Version 10.6.0 software.

Unsupervised clustering was performed on the expression values of the markers using the FlowSOM algorithm (R package CATALYST, version 1.14.0) (61, 62), which uses a self-organizing map followed by hierarchical consensus metaclustering to detect cell populations (63). In each sample, 50,000 randomly selected, intact, live PBMCs, all cells within the NK cells gate, or all cells within Th2 cells gate were projected into the clustering analysis, and the marker expression values were Arcsinh transformed with a cofactor of 5. Default parameters and a predetermined number of 40 clusters (k = 40) were used for total PBMC clustering analysis, 15 clusters (k = 15) for total NK cell clustering analysis and 15 clusters (k = 15) for total memory Th2 cell clustering analysis. The median levels of the lineage markers across all cells per cluster were visualized in a heatmap (R package ComplexHeatmap, version 2.6.2) (64). The main subtypes of immune cells were identified based on the median expression levels of markers in each cluster. We applied the nonlinear dimensionality reduction technique (UMAP) to detect the expression levels of marker from a set of randomly selected cells using the R package UMAP (R package CATALYST, version 1.14.0, default parameters).

### Flow cytometry.

The antibodies used for flow cytometry staining are listed in Supplemental Table 4. Cells were collected after 3 days of culture. Cell staining was performed as previously described (20). For CD107a staining, the cultured cells were incubated in the presence of anti-CD107a antibody and 10 nM monensin during the last 4 hours of the cell culture. All samples were run on Aurora spectral flow cytometry and analyzed using SpectroFlo software and FlowJo Version 10.8.0 software.

### Cytokine assays.

The supernatants were harvested from the 3-day culture of mock-depleted PBMCs, CD3^+^ T cell–depleted PBMCs, or CD56^+^ cell–depleted PBMCs in the presence or absence of peanut protein. The secretion level of cytokines from PBMCs in supernatants were measured using the Luminex 200 IS system (Affymetrix) by Stanford’s Human Immune Monitoring Center (HIMC) and the MFI value for each analyte per sample was reported by Stanford’s HIMC.

### Statistics.

Statistical comparison analyses were performed using Wilcoxon test function in R software. We compared different in vitro treatments from the same participant using Wilcoxon signed rank comparisons test (nonparametric paired test, 2 sided) and the same treatments in vitro in PA participants and NA participants using Wilcoxon rank sum comparisons test (nonparametric unpaired test, 2 sided). The percent change in cell frequency was calculated as the difference between the average values with or without peanut stimulation. The degree of increase in the CD69^+^ NK cells in response to peanut stimulation was calculated by subtracting the percentage of this population in an unstimulated sample from that of the corresponding stimulated sample. For these comparison analyses of cell subset frequencies obtained from FlowSOM-based unsupervised clustering analyses, *P* values were adjusted for multiple comparisons using the Bonferroni approach to control the FDR, and FDR-adjusted *P* < 0.05 was considered significant.

PCA was performed using FactoMineR and factoextra R package. Euclidean distances were performed using distance function in R. Using “createDataPartition” function in caret R package, the mass cytometry results were split randomly into a 70% training set for training the regularized (penalized) logistic regression model and RF model, and the remaining 30% into a test set to evaluate the model performance. Regularized (penalized) logistic regression was performed using the “glmnet” R package (28). The RF algorithm was performed using the “RandomForest” R package. Receiver operating characteristic (ROC) analysis was performed using “pROC” R Package. All dot plots overlaid with boxplots, line connections or violin plots were compiled with ggplot2 R package or Prism 8 (GraphPad).

### Study approval.

All studies were approved by the Institutional Review Board of Stanford University (ClinicalTrials.gov NCT02103270, NCT01613885, and NCT02626611.). Informed consent was obtained from all patients or their caregivers.

## Author contributions

The authorship order among co–first authors was based on XZ having designed and performed all experiments and outlined the manuscript. WY wrote much of the manuscript and revised the manuscript. XZ, WY, CAB, RHD, and KCN conceptualized the project. XZ, WY, DMD, and JPS were responsible for methodology. XZ, WY, RHD, and KCN performed the investigations. RHD and KCN supervised the project. XZ and WY wrote the original draft. XZ, WY, CAB, RHD, and KCN reviewed and edited the manuscript.

## Supplementary Material

Supplemental data

Supplemental tables 1-6

## Version 1 10/17/2022

Electronic publication
