# Supplementary material for: Cytometric analysis reveals an association between allergen-responsive natural killer cells and human peanut allergy
Source: J Clin Invest. 2022 Oct 17;132(20):e157962. doi: 10.1172/JCI157962 (PMC9566921; doi:10.1172/JCI157962)
Supplement: Supplemental data [file jci-132-157962-s111.pdf]

Supplementary Materials for

Cytometric analysis reveals an association between allergen-responsive natural killer cells and human peanut allergy

Authors: Xiaoying Zhou[1†], Wong Yu[1,2†], Diane M. Dunham[1], Jackson P. Schuetz[1], Catherine A. Blish[3], Rosemarie H. DeKruyff [‡1], Kari C. Nadeau[1‡*]

Affiliations: [1]Sean N. Parker Center for Allergy & Asthma Research at Stanford University and Division of Pulmonary, Allergy, and Critical Care Medicine, Stanford, CA, 94305, USA

[2] Division of Allergy, Immunology and Blood and Marrow Transplantation, Department of Pediatrics, University of California, San Francisco

[3] Program in Immunology and Department of Medicine, Stanford University School of Medicine, Stanford, CA 94305, USA

***Corresponding author**

Kari C. Nadeau, MD, PhD, Director, Sean N. Parker Center for Allergy and Asthma Research at Stanford University, 240 Pasteur Dr. BMI Rm.1755, Palo Alto, CA 94304, USA. Phone number: 650-468-1236. E-mail: knadeau@stanford.edu. ORCID: 0000-0002-2146-2955.

†These authors contributed equally to this work as first authors.
‡These authors contributed equally to this work as senior authors.

This PDF includes:

Fig. S1. FlowSOM-based clustering analysis of PBMCs from peanut allergic vs nonallergic subjects cultured with or without peanut protein.

Fig. S2. Frequencies of the eleven Th2 cell subsets identified in Fig. 2.

Fig. S3: Cytokine expression in activated ($CD69^+$) NK cells.

Fig. S4. Cytokine expression in activated ($CD69^+$) EM Th2 cells and activated ($CD69^+$) NK cells.

Fig. S5. The Lasso logistic regression and Random Forest classification models.

Fig. S6. Median intensity of CD56 and CD69 in NK cells and Median fluorescence intensity (MFI) of CD16, NKp46, NKG2C, KIR and perforin in NK cells were examined using mass cytometry and Aurora spectrum flow cytometry, respectively.

Fig. S7. NK cell receptors (Nkp30, Nkp46, NKG2C and KIR [KIR2DL2/L3/KIR3DL1]), a functional activity marker (CD107a) and a lytic activity marker (perforin)were examined using Aurora spectrum flow cytometry.

Fig. S8. Surface marker expression of NK cells from PBMCs of PA and NA participants cultured with peanut protein or media.

Fig. S9. CD86 expression in NK cells, B cells and myeloid cells.

Fig. S10. FlowSOM based clustering analysis of NK cells from mock-depleted PBMCs and $CD3^+$ T cell-depleted PBMCs in PA participants cultured with peanut protein or media.

Fig. S11. The secretion of cytokines in supernatant from mock depleted PBMCs, $CD3^+$ T cell-depleted PBMCs and $CD56^+$ cell-depleted PBMCs.

Fig. S12. OIT is associated with a decreased expansion of NK cells after peanut stimulation.

Tables S1 to S6 are provided as Supplementary Data File in Excel Spreadsheet format.

Supplementary Figure 1

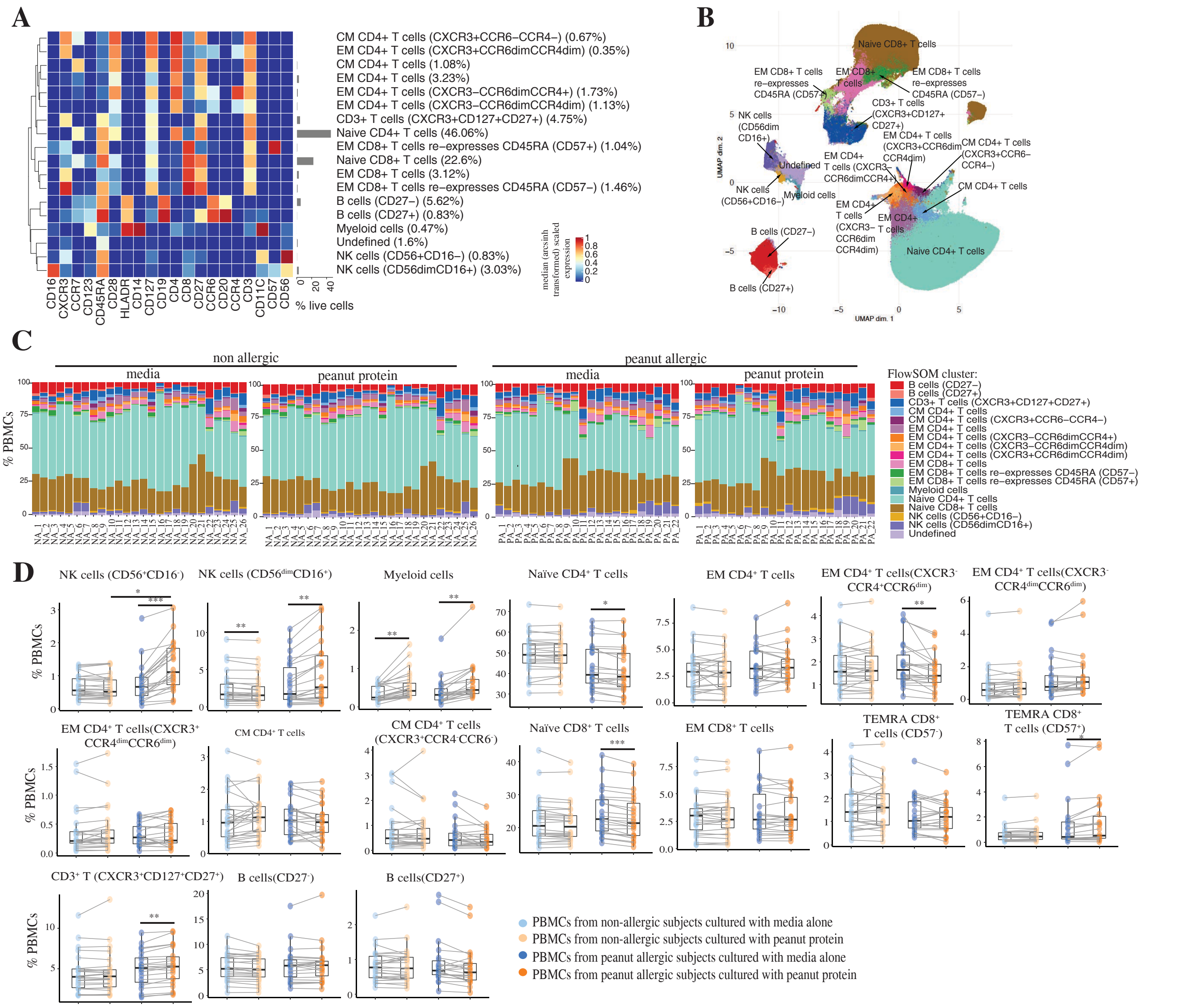

Supplementary Figure 1: FlowSOM-based clustering analysis of PBMCs from peanut allergic vs nonallergic subjects cultured with or without peanut protein.

(A) FlowSOM based clustering analysis was performed on total 4,800,000 cells (50,000 cells per sample; total 96 samples: n=26 for NA PBMCs cultured with or without peanut protein, n=22 for PA PBMCs cultured with or without peanut protein) randomly selected from pre-gated live single PBMCs cultured with either media alone or peanut protein from NA and PA participants. Heatmap display the median expression levels of cell surface markers in each cluster. The marker expression values were Arcsinh (inverse hyperbolic sine) transformed with a cofactor of 5. The bar graph shows each cluster as percentage of live cells. **(B)** Uniform Manifold Approximation and Projection (UMAP) representation of 960,000 randomly selected cells (10,000 per sample; total 96 samples: n=26 for NA PBMCs cultured with or without peanut protein, n=22 for PA PBMCs cultured with or without peanut protein); clusters from the FlowSOM analysis are indicated by color. **(C)** Stacked bar graph visualizing the frequencies of clusters identified by the FlowSOM analysis in individual participants using the color-code indicated on the bottom (n=26 for NA PBMCs cultured with or without peanut protein, n=22 for PA PBMCs cultured with or without peanut protein). **(D)** The frequencies of 17 cell subsets in total PBMCs stimulated with or without peanut protein for NA (n=26) and PA (n=22) participants are shown in the box plots overlaid with dot plots. Each pair of points connected by a line represents one subject. Box plots indicate the interquartile range (IQR) and median; whiskers extend to the farthest data point within a maximum of 1.5× IQR. Paired sample sets were analyzed using the Wilcoxon signed rank test (two sided). Unpaired sample sets were analyzed using the Wilcoxon rank sum test (two sided). P-values were adjusted for multiple comparisons using Bonferroni approach to control the FDR. FDR-adjusted p < 0.05 were considered significant. The stars indicate the FDR-adjusted P values: ***P < 0.001, **P < 0.01, and *P < 0.05.

Supplementary Figure 2

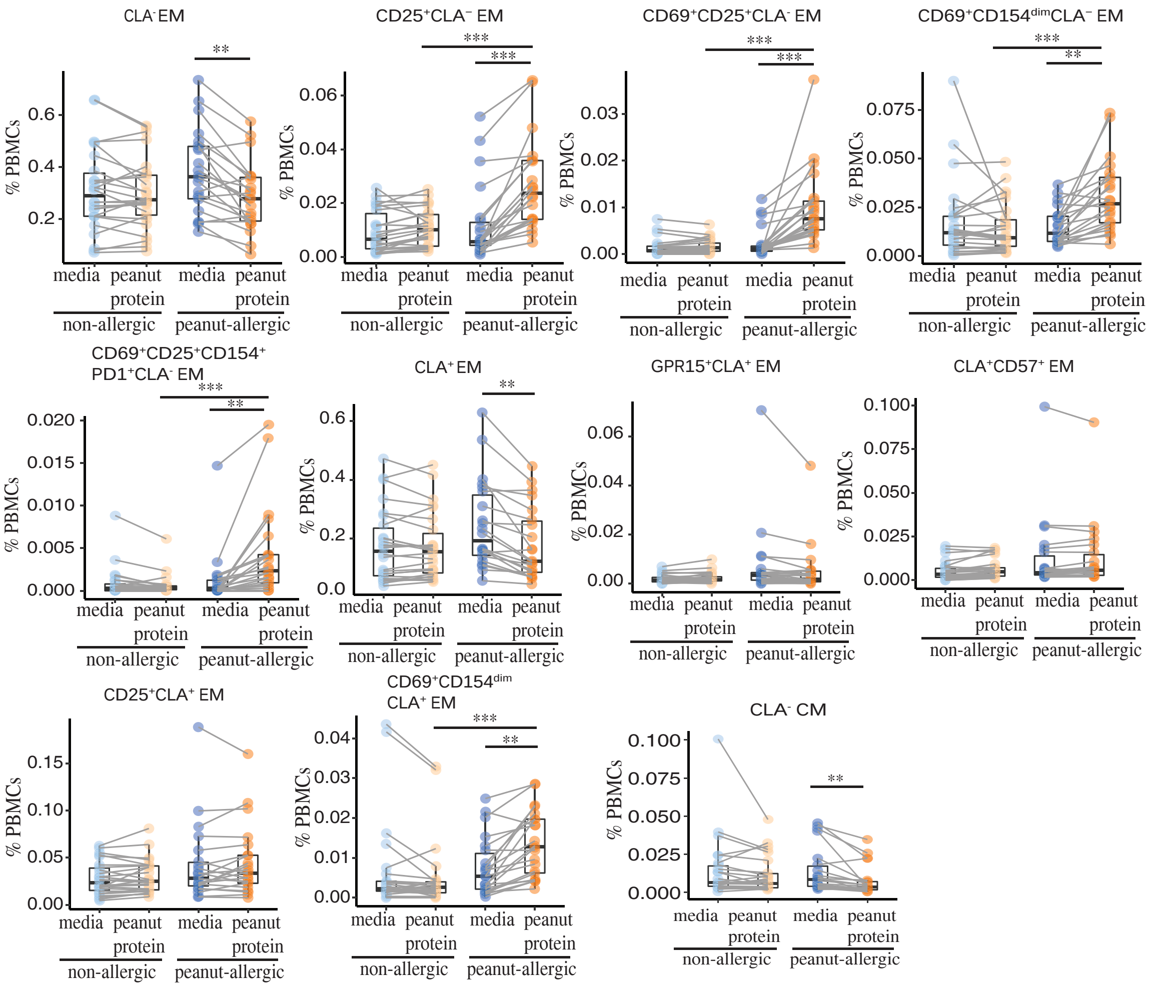

Supplementary Figure 2: The frequencies of the eleven Th2 cell subsets identified in Fig. 2.

The frequencies of the eleven Th2 cell subsets in total PBMCs from NA (n=26) and PA (n=22) participants cultured with or without peanut stimulation are shown in the box plots overlaid with dot plots. Each pair of points connected by a line represents one subject. Box plots indicate the interquartile range (IQR) and median; whiskers extend to the farthest data point within a maximum of 1.5× IQR. Paired sample sets were analyzed using the Wilcoxon signed rank test (two sided). Unpaired sample sets were analyzed using the Wilcoxon rank sum test (two sided). P-values were adjusted for multiple comparisons using the Bonferroni approach to control the FDR. FDR-adjusted $p < 0.05$ were considered significant. The stars indicate the FDR-adjusted P values: ***$P < 0.001$, **$P < 0.01$, and *$P < 0.05$.

Supplementary Figure 3

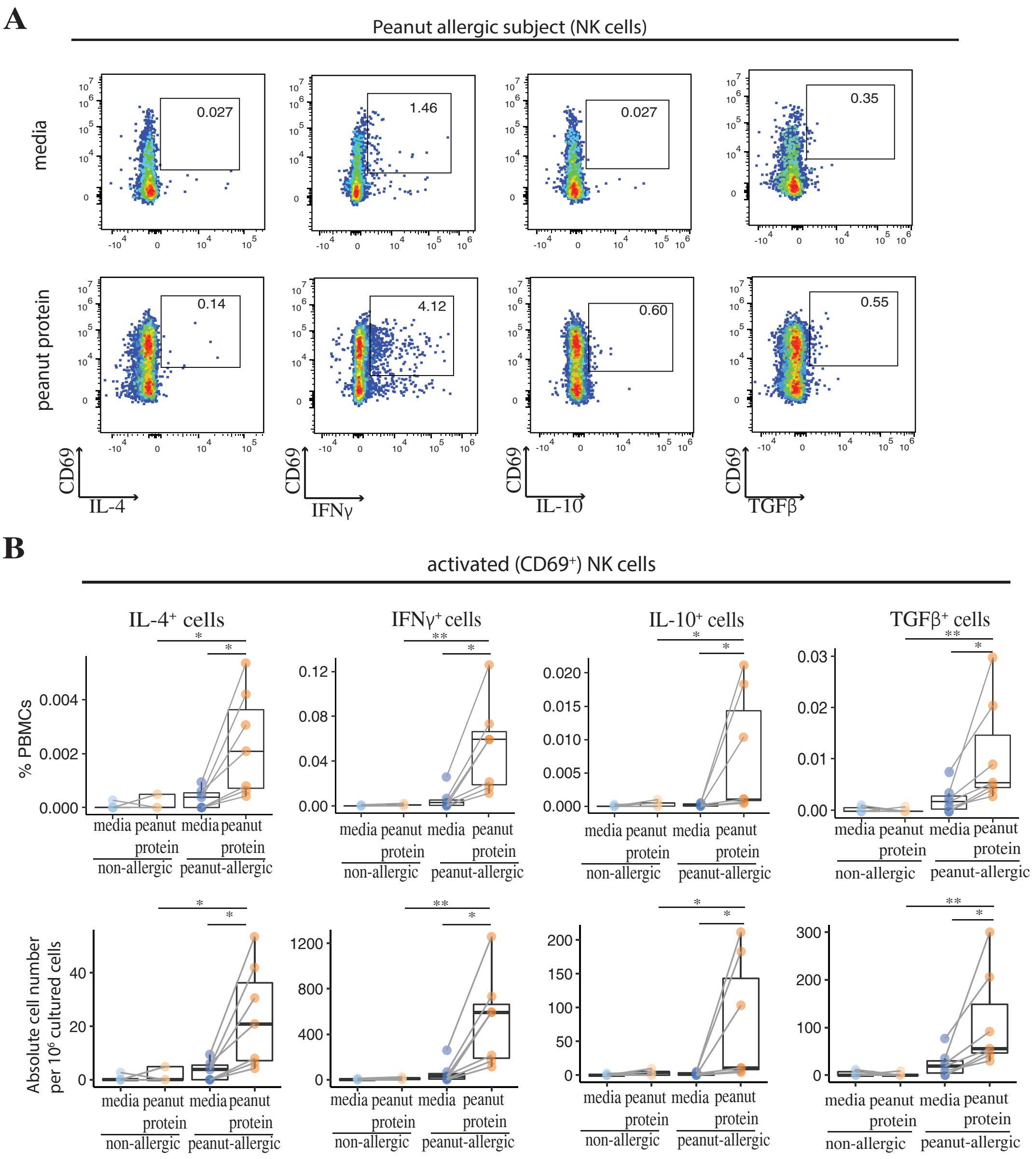

Figure. S3. Cytokine expression in activated (CD69⁺) NK cells.

(A) Representative flow cytometry plots show the gating strategy for cytokine (IL-4, IFN-γ, IL-10 and TGF-β)-expressing CD69⁺ NK cells. **(B)** Frequencies of cytokine-expressing CD69⁺ NK cells expressed as a percentage of total PBMCs (top) and their absolute number per 10^6 cultured cells (bottom) from NA (n=5) and PA (n=7) participants with or without peanut stimulation. Each pair of points connected by a line represents one subject. Box plots indicate the interquartile range (IQR) and median; whiskers extend to the farthest data point within a maximum of 1.5× IQR. Paired sample sets were analyzed using the Wilcoxon signed rank test (two sided). Unpaired sample sets were analyzed using the Wilcoxon rank sum test (two sided). *P<0.05, **P<0.01.

Supplementary Figure 4

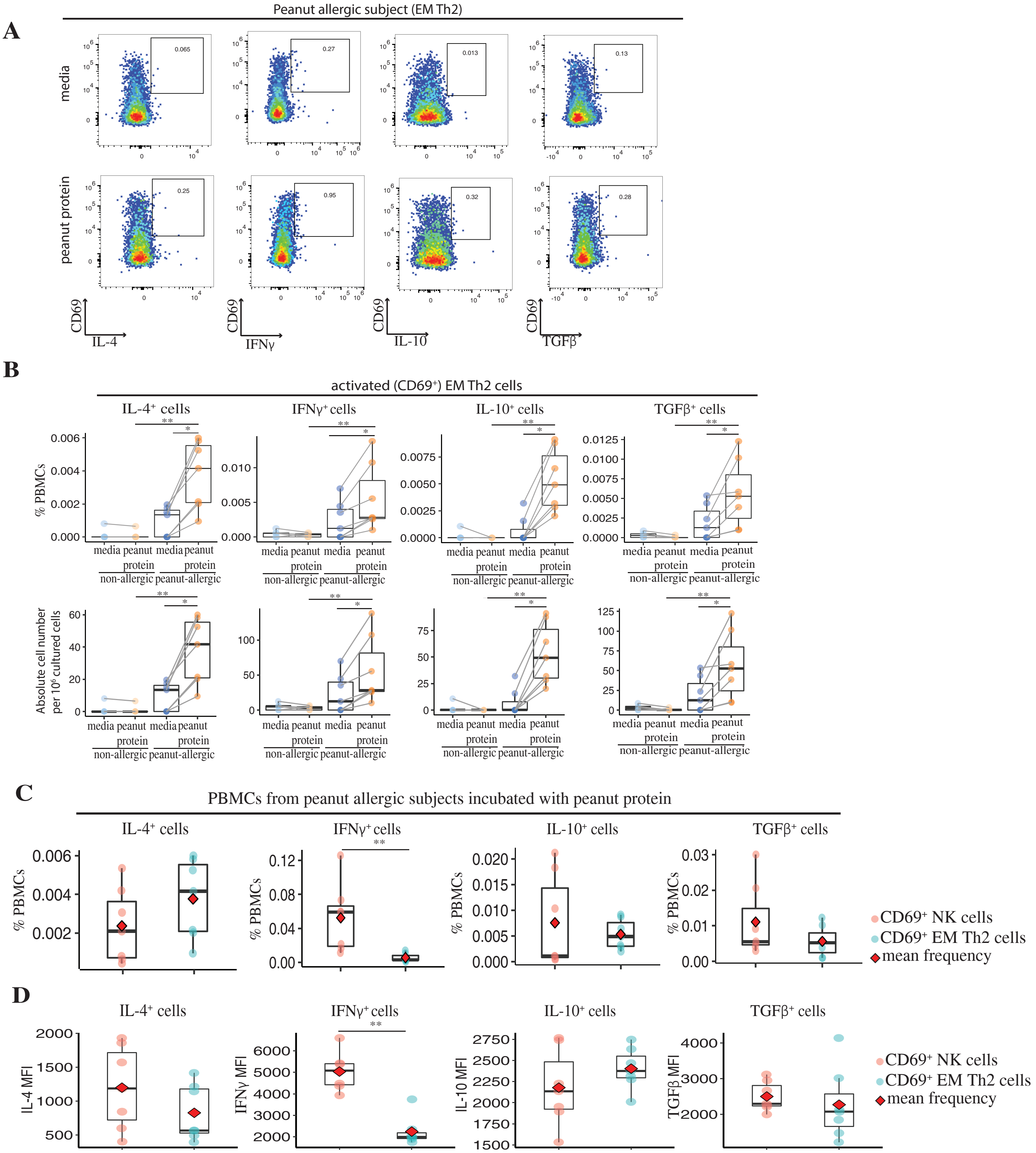

Figure. S4. Cytokine expression in activated ($CD69^+$) EM Th2 cells and activated ($CD69^+$) NK cells.

(A) Representative flow cytometry plots show the gating strategy for cytokine (IL-4, IFN-γ, IL-10 and TGF-β)-expressing $CD69^+$ EM Th2 cells. **(B)** Frequencies of cytokine-expressing $CD69^+$ EM Th2 cells expressed as a percentage of total PBMCs (top) and their absolute number per 10^6 cultured cells (bottom) from NA (n=5) and PA participants (n=7) with or without peanut stimulation. Each pair of points connected by a line represents one subject. **(C)** Comparison of the frequency of cytokine expression (IL-4, IFN-γ, IL-10 and TGF-β) between activated NK cells (Figure. S3) and activated EM Th2 cells following peanut protein stimulation in PA subjects (n=7). **(D)** Comparison of cytokine median fluorescence intensity (MFI) values in cytokine-expressing activated NK cells and activated EM Th2 cells following peanut protein stimulation in PA subjects (n=7). Each point represents one subject. The red rhombus represents the mean frequency. Box plots indicate the interquartile range (IQR) and median; whiskers extend to the farthest data point within a maximum of 1.5× IQR. Paired sample sets were analyzed using the Wilcoxon signed rank test (two sided). Unpaired sample sets were analyzed using the Wilcoxon rank sum test (two sided). **$P<0.01, *$P<0.05$.

Supplementary Figure 5

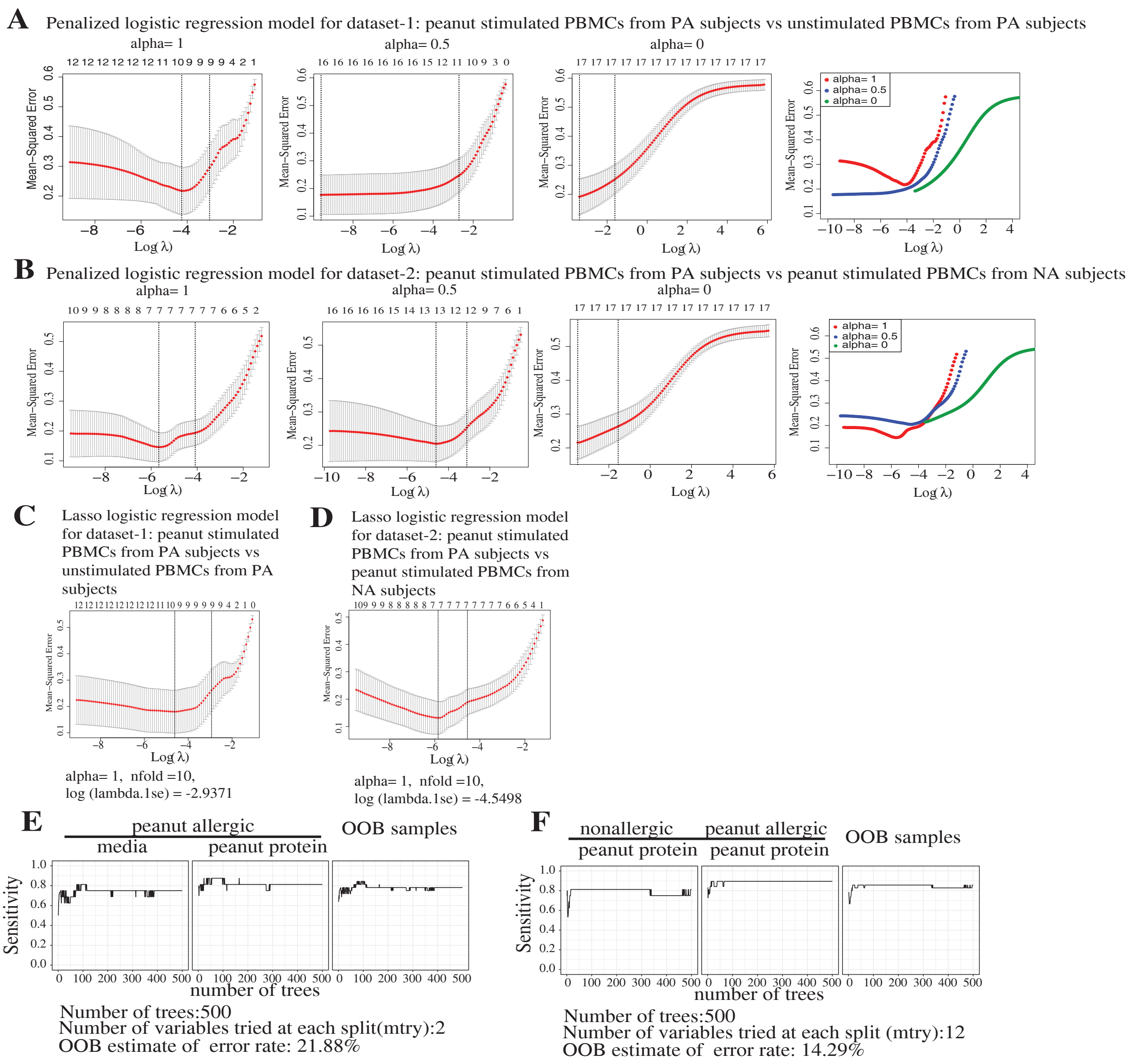

Fig. S5. The Lasso logistic regression and Random Forest classification models.

(A and B) The estimate of the mean squared error for lasso (alpha=1), elastic (alpha= 0.5) or ridge (alpha=0) models with explicitly controlling the fold of cross-validation for dataset-1with 17 variables (cell subsets) and 44 observations (22 PA subjects with or without peanut stimulation) (A) and dataset-2 with 17 variables (cell subsets) and 48 observations (peanut stimulated 22 PA subjects and peanut stimulated 26 NA subjects) (B). **(C and D)** The lasso logistic regression (alpha =1) with 10-fold cross validation is performed for dataset-1(peanut stimulated PBMCs from PA subjects vs. unstimulated PBMCs from PA subjects) (C) and dataset-2 (peanut stimulated PBMCs from PA subjects vs. peanut stimulated PBMCs from NA subjects) (D). The dashed lines show the locations of λ that give the cross-validated error within one standard error of the minimum (lambda.1se) and the minimum cross-validated error (lambda.min). **(E and F)** Plots show the sensitivity with the number of trees (ntree) for the RF models in dataset-1(peanut stimulated PBMCs from PA subjects vs. unstimulated PBMCs from PA subjects) (E) and dataset-2 (peanut stimulated PBMCs from PA subjects vs. peanut stimulated PBMCs from NA subjects) (F).

Supplementary Figure 6

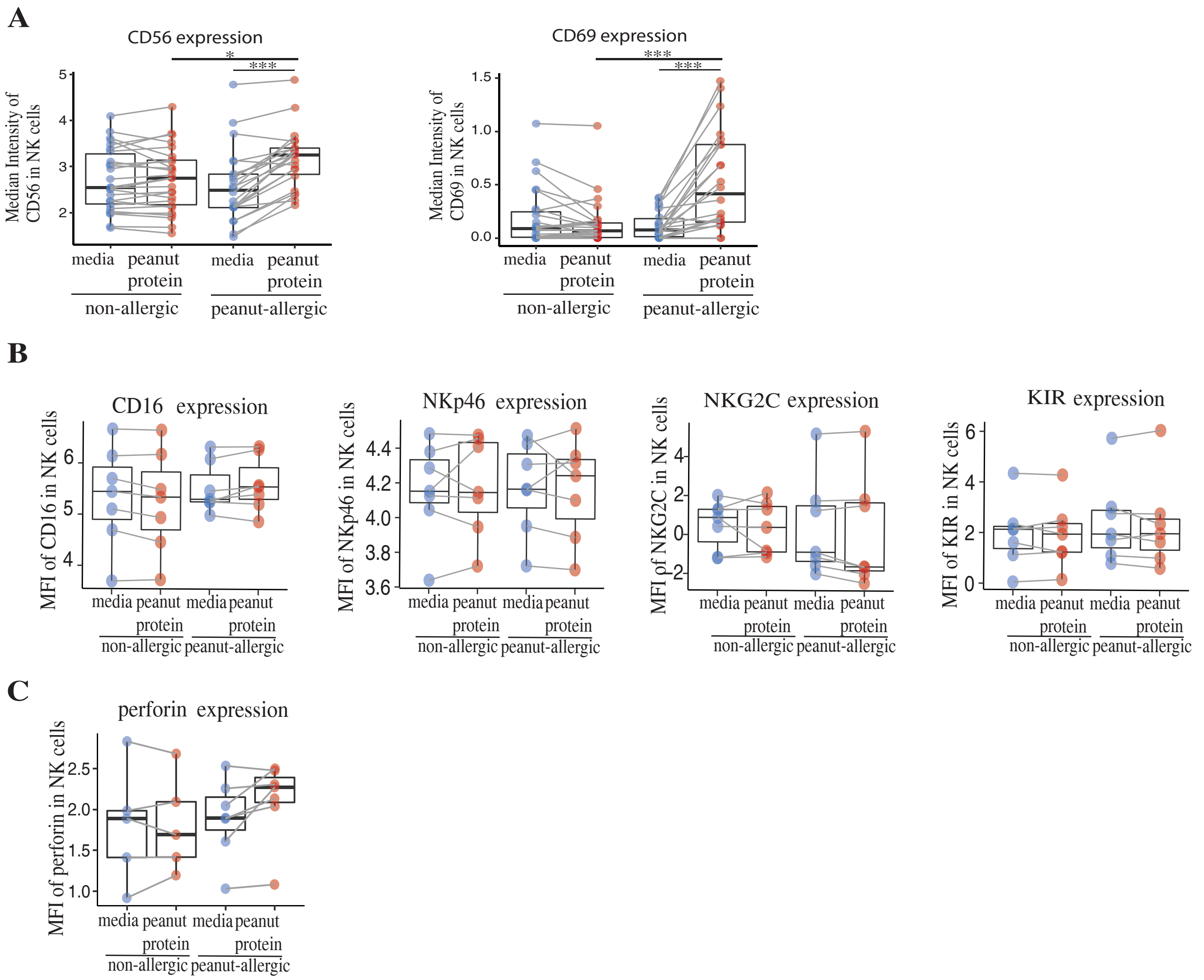

Figure. S6. Median intensity of CD56 and CD69 in NK cells and Median fluorescence intensity (MFI) of CD16, NKp46, NKG2C, KIR and perforin in NK cells were examined using mass cytometry and Aurora spectrum flow cytometry, respectively.

(A) Median intensity (MFI) of CD56 and CD69 in NK cells in NA (n=26) and PA (n=22) samples stimulated with or without peanut protein. The arcsinh transformation using standard cofactor 5 was applied for the expression value of each marker. **(B)** Median fluorescence intensity (MFI) of CD16, NKp46, NKG2C and KIR in NK cells in NA (n=7) and PA (n=7) samples stimulated with or without peanut protein. **(C)** MFI of perforin in NK cells in NA (n=5) and PA (n=7) samples stimulated with or without peanut protein.The arcsinh transformation using standard cofactor 150 was applied for the expression value of each marker. Box plots indicate the interquartile range (IQR) and median; whiskers extend to the farthest data point within a maximum of 1.5× IQR. Paired sample sets were analyzed using the Wilcoxon signed rank test (two sided). Unpaired sample sets were analyzed using the Wilcoxon rank sum test (two sided). *P<0.05, ***P<0.001.

Supplementary Figure 7

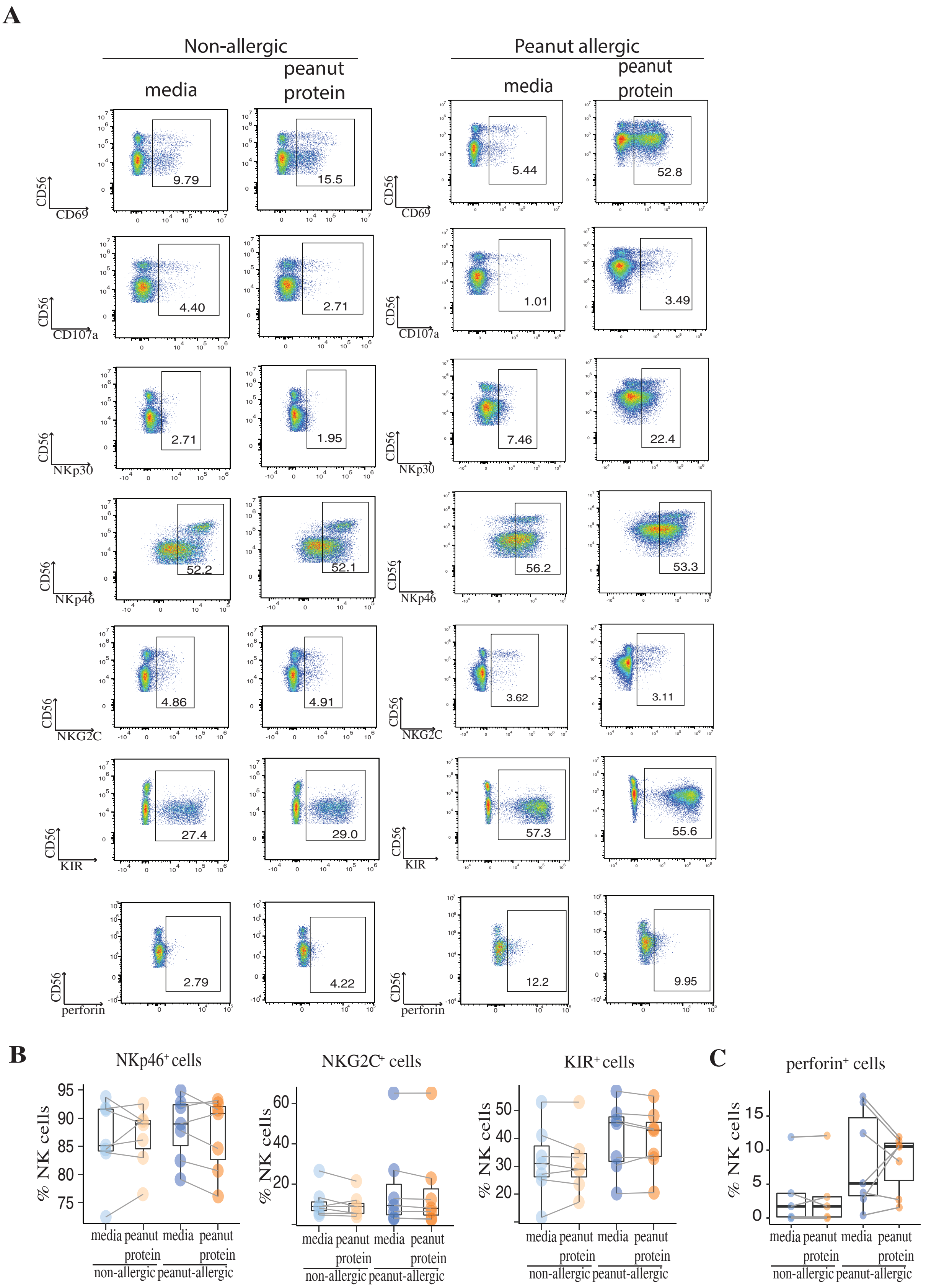

Figure. S7. NK cell receptors (Nkp30, Nkp46, NKG2C and KIR [KIR2DL2/L3/KIR3DL1]), a functional activity marker (CD107a) and and a lytic activity marker (perforin) were examined using Aurora spectrum flow cytometry.

(A) Representative flow cytometry plots gated on NK cells show the CD69^{+} NK cells, CD107a^{+} NK cells, NKp30^{+} NK cells, NKp46^{+} NK cells, NKG2C^{+} NK cells, KIR^{+} NK cells and perforin^{+} NK cells **(B)** Frequencies of the NKp46^{+} NK cells, NKG2C^{+} NK cells and KIR^{+} NK cells among NK cell population cultured with or without peanut protein from NA (n=7) vs PA participants (n=7). **(C)** Frequencies of the perforin^{+} NK cells among NK cell population cultured with or without peanut protein from NA (n=5) vs PA participants (n=7). Box plots indicate the interquartile range (IQR) and median; whiskers extend to the farthest data point within a maximum of 1.5× IQR. Paired sample sets were analyzed using the Wilcoxon signed rank test (two sided). Unpaired sample sets were analyzed using the Wilcoxon rank sum test (two sided).

Supplementary Figure 8

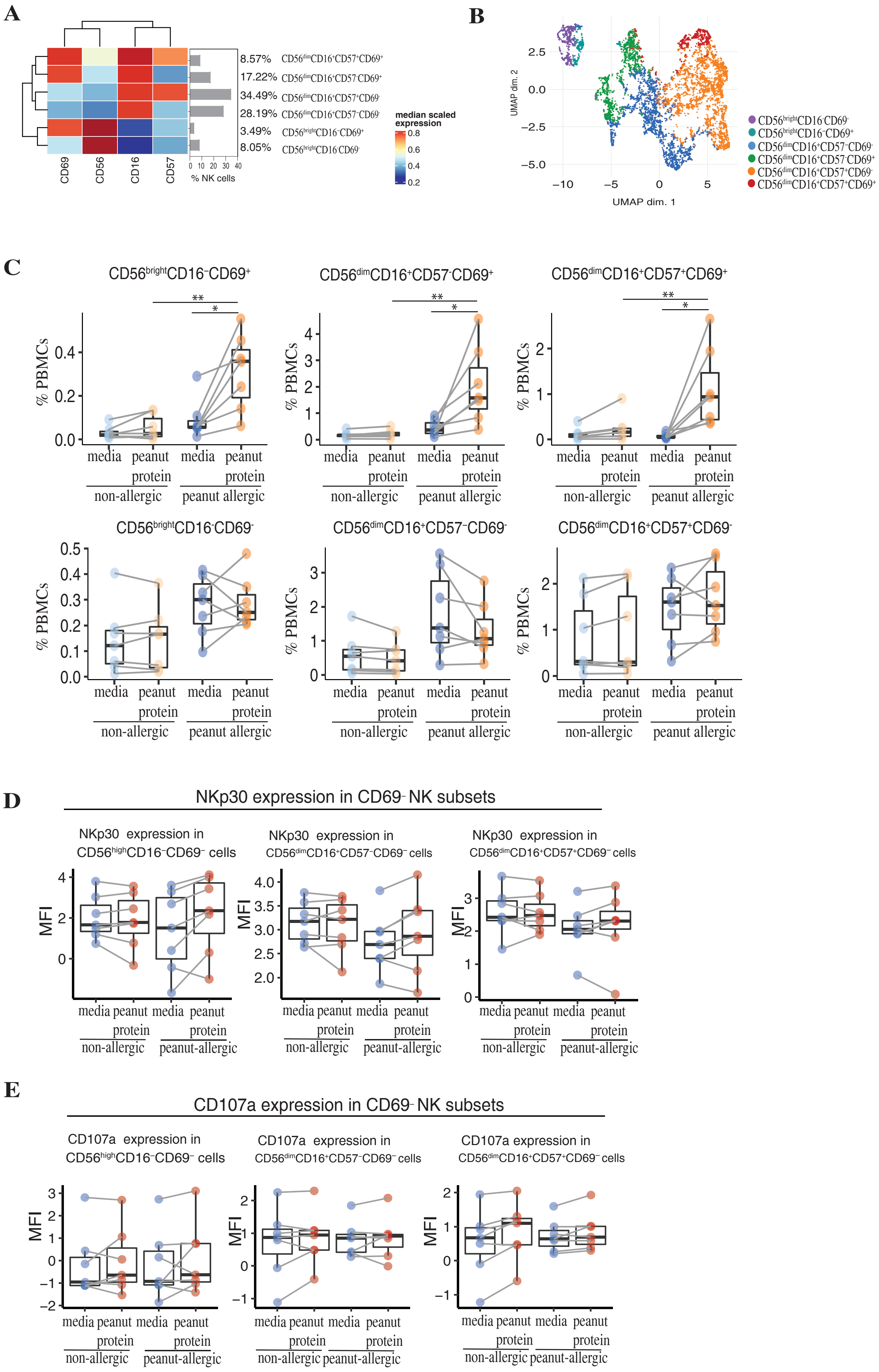

Figure. S8. Surface marker expression of NK cells from PBMCs of PA and NA participants cultured with peanut protein or media.

(A) FlowSOM based clustering analysis of data acquired on Aurora spectral flow cytometer was performed on gated NK cells from PBMCs in NA (n=7) and PA (n=7) subjects cultured with either media alone or peanut protein. Heatmap representing the median expression levels of cell surface markers in each cluster. The marker expression values were arcsinh transformed with a cofactor of 150. The bar graph shows each cluster as percent of NK cells. **(B)** UMAP representation of 7,000 randomly selected cells (250 cells per sample; total 28 samples in 4 experimental groups: PA vs NA, each with or without peanut protein stimulation), clusters from the FlowSOM analysis are indicated by color. **(C)** The frequencies of six NK cell subsets as a percentage of total PBMCs from NA (n=7) and PA (n=7) participants with or without peanut stimulation. **(D and E)** MFI of NKp30 (D) and CD107a (E) on $CD69^-$ NK subsets ($CD69^-CD56^{high}CD16^-$, $CD69^-CD56^{dim}CD16^+CD57^-$ and $CD69^-CD56^{dim}CD16^+CD57^+$) cultured with or without peanut protein from NA (n=7) vs PA participants (n=7). Box plots indicate the interquartile range (IQR) and median; whiskers extend to the farthest data point within a maximum of 1.5× IQR. Paired sample sets were analyzed using the Wilcoxon signed rank test (two sided). Each pair of points connected by a line represents one subject. Paired sample sets were analyzed using the Wilcoxon signed rank test (two sided). Unpaired sample sets were analyzed using the Wilcoxon rank sum test (two sided). *P<0.05, **P<0.01.

Supplementary Figure 9

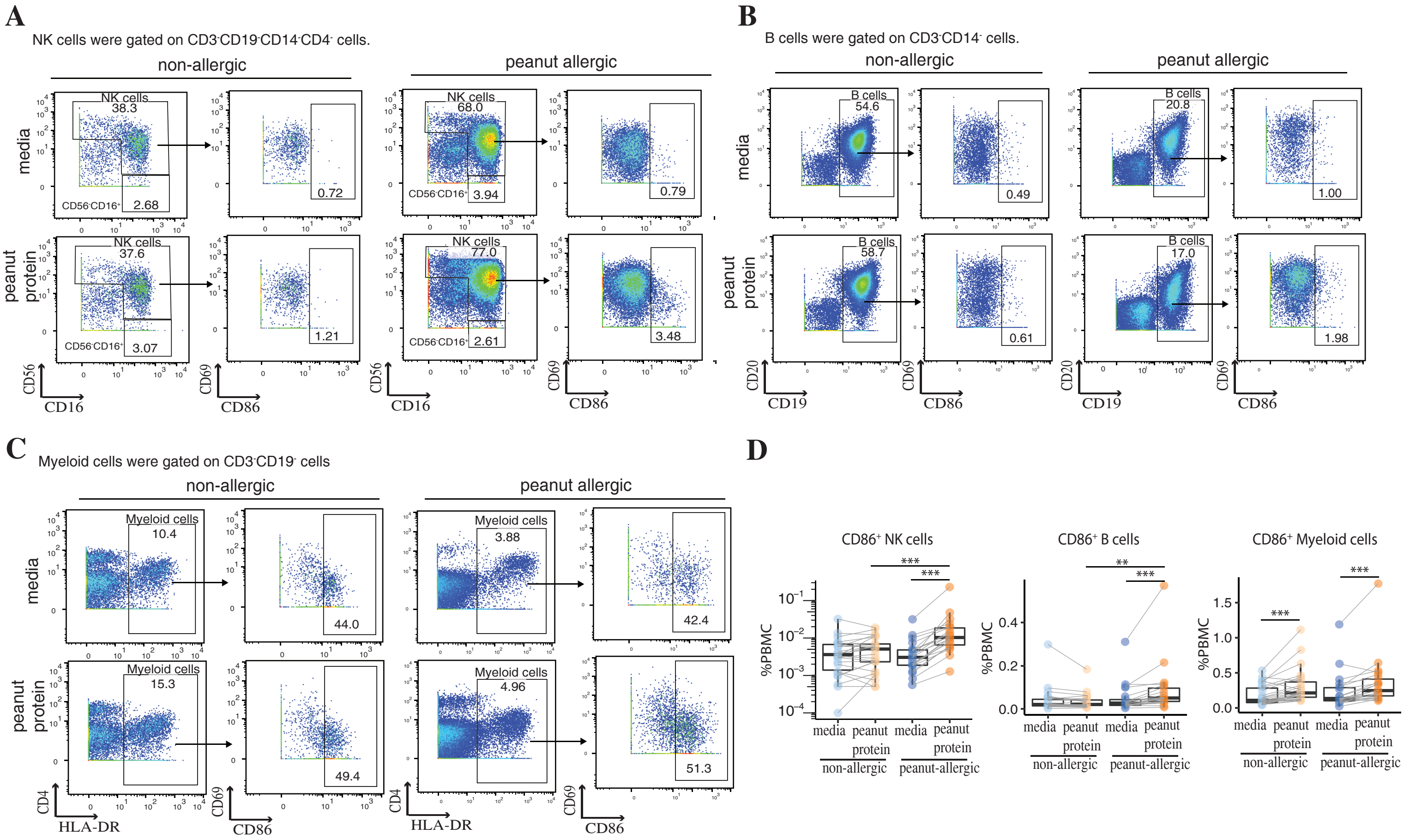

Figure S9. CD86 expression in NK cells, B cells and myeloid cells.

(**A-C**) Mass cytometry plots show the gating strategy for CD86+ cells in NK cells (NK cells includes both the CD56dimCD16+ and the CD56brightCD16– NK subpopulations gated on CD14-CD19-CD3-CD4- cells) (A), B cells (CD3-CD14-CD19+) (B) and myeloid cells (CD3-CD19-HLA-DR+) (C). (**D**) Frequencies of the CD86-expressing NK cells (left), B cells (middle) and myeloid cells (right) in total PBMCs cultured with or without peanut protein from NA (n=26) vs PA (n=22) participants. Each pair of points connected by a line represents one subject. Paired sample sets were analyzed using the Wilcoxon signed rank test (two sided). Unpaired sample sets were analyzed using the Wilcoxon rank sum test (two sided). **P<0.01, ***P<0.001.

Supplementary Figure 10

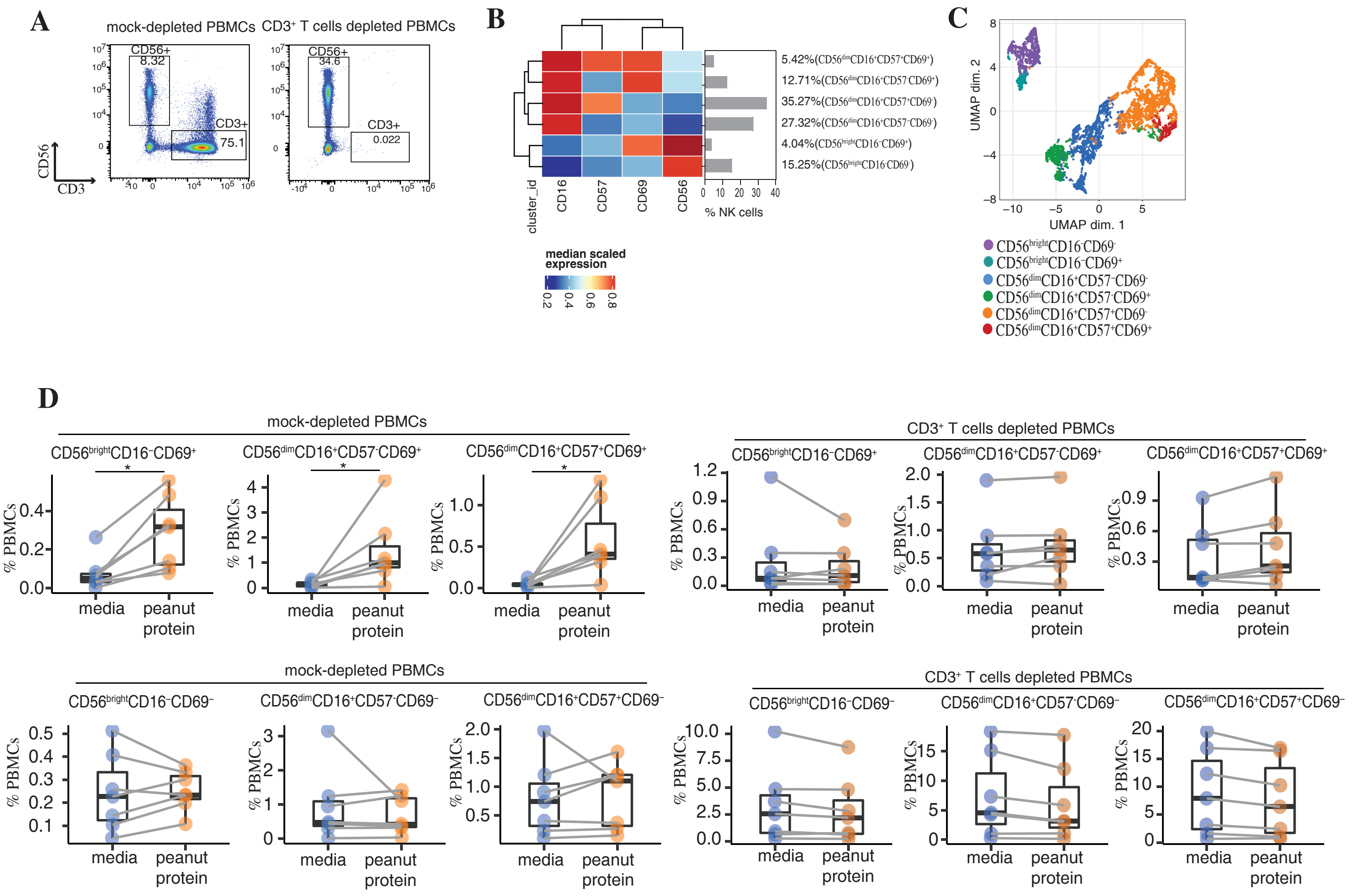

Figure S10. FlowSOM based clustering analysis of NK cells from mock-depleted PBMCs and CD3+ T cell-depleted PBMCs in PA participants cultured with peanut protein or media.

(**A**) Flow cytometry plots show the gating for CD56+ cells and CD3+ cells in mock-depleted PBMCs and CD3+ T cell-depleted PBMCs. The mock-depleted sample was the PBMCs from the same PA subjects were treated identically in parallel but without addition of CD3 microbeads. (**B**) FlowSOM-based clustering analysis was performed on gated NK cells from mock-depleted PBMCs and CD3+ T cell-depleted PBMCs from PBMCs in 7 PA subjects cultured with either media alone or peanut protein. Heatmap representing the median expression levels of cell surface markers in each cluster. The marker expression values were arcsinh transformed with a cofactor of 150. The bar graph shows each cluster as as percentage of NK cells. (C) UMAP representation of 7,000 randomly selected cells (250 cells per sample, total 28 samples in 4 experimental groups: mock-depleted PBMCs vs CD3+ T cell-depleted PBMCs, each with or without peanut protein stimulation), clusters from the FlowSOM analysis are indicated by color. (D) The frequencies of six NK cell subsets as a percentage of mock-depleted PBMCs (left) or CD3+ T cell-depleted PBMCs (right) from 7 PA participants with or without peanut stimulation.Each pair of points connected by a line represents one subject. Paired sample sets were analyzed using the Wilcoxon signed rank test (two sided). Unpaired sample sets were analyzed using the Wilcoxon rank sum test (two sided). *P<0.05.

Supplementary Figure 11

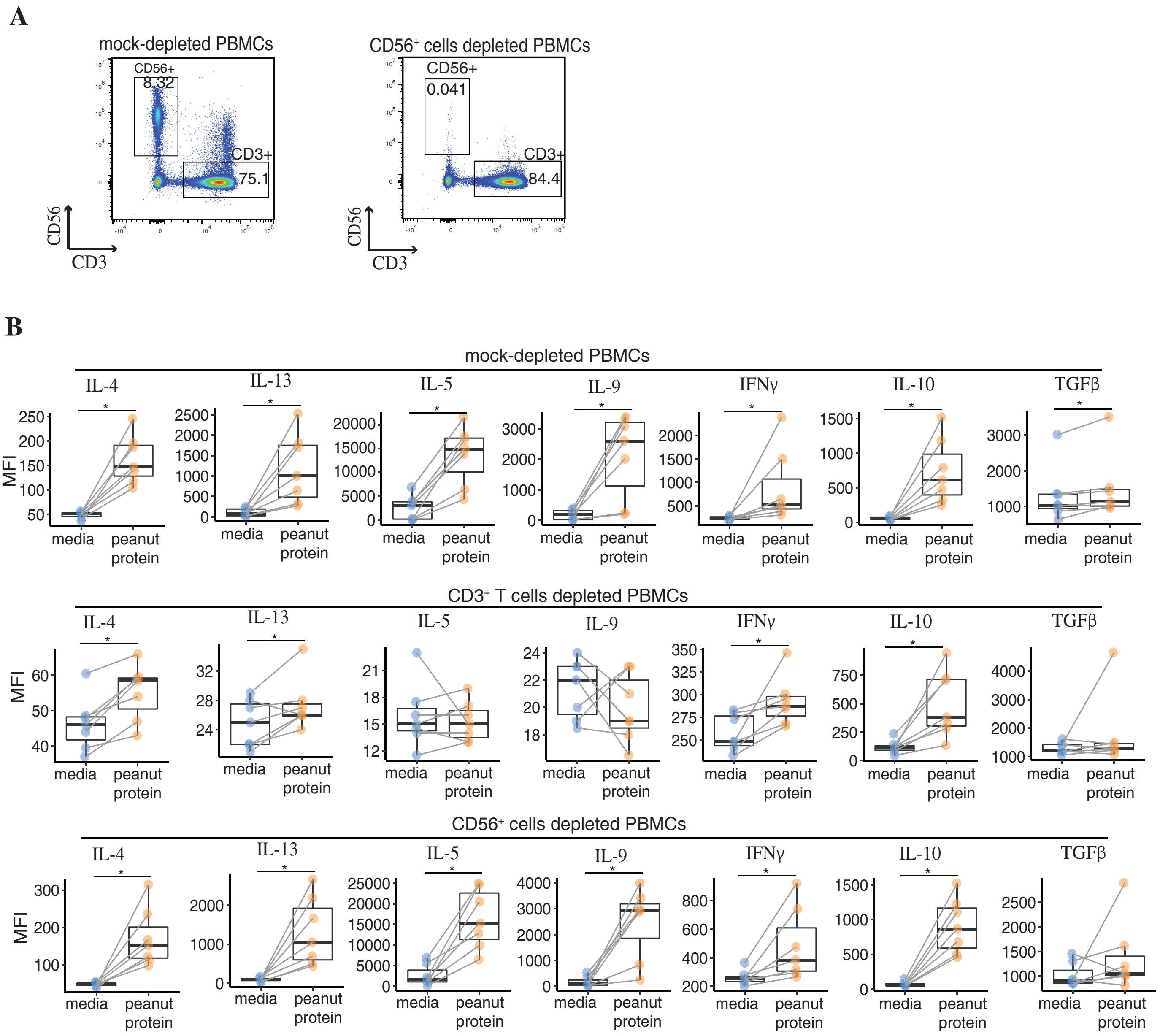

Figure S11. The secretion of cytokines in supernatant from mock depleted PBMCs, CD3⁺ T cell-depleted PBMCs and CD56⁺ cell-depleted PBMCs.

(A) Flow cytometry plots show the gating for CD56⁺ cells and CD3⁺ cells in mock-depleted PBMCs and CD56⁺ cell-depleted PBMCs.**(B)** The secretion of cytokines (Type 2 cytokines [IL-4, IL-13, IL-9, IL-5], Type 1 cytokine IFN-γ, and immunoregulatory cytokines [IL-10 and TGF-β]) measured by Luminex based assay in mock-depleted PBMCs (top), CD3⁺ T cell-depleted PBMCs (middle) and CD56⁺ cell-depleted PBMCs (bottom) stimulated with or without peanut protein. Please note the different y axes in upper vs middle panel. Box plots indicate the interquartile range (IQR) and median; whiskers extend to the farthest data point within a maximum of 1.5× IQR. Paired sample sets were analyzed using the Wilcoxon signed rank test (two sided). Each pair of points connected by a line represents one subject. *$P<0.05$.

Supplementary Figure 12

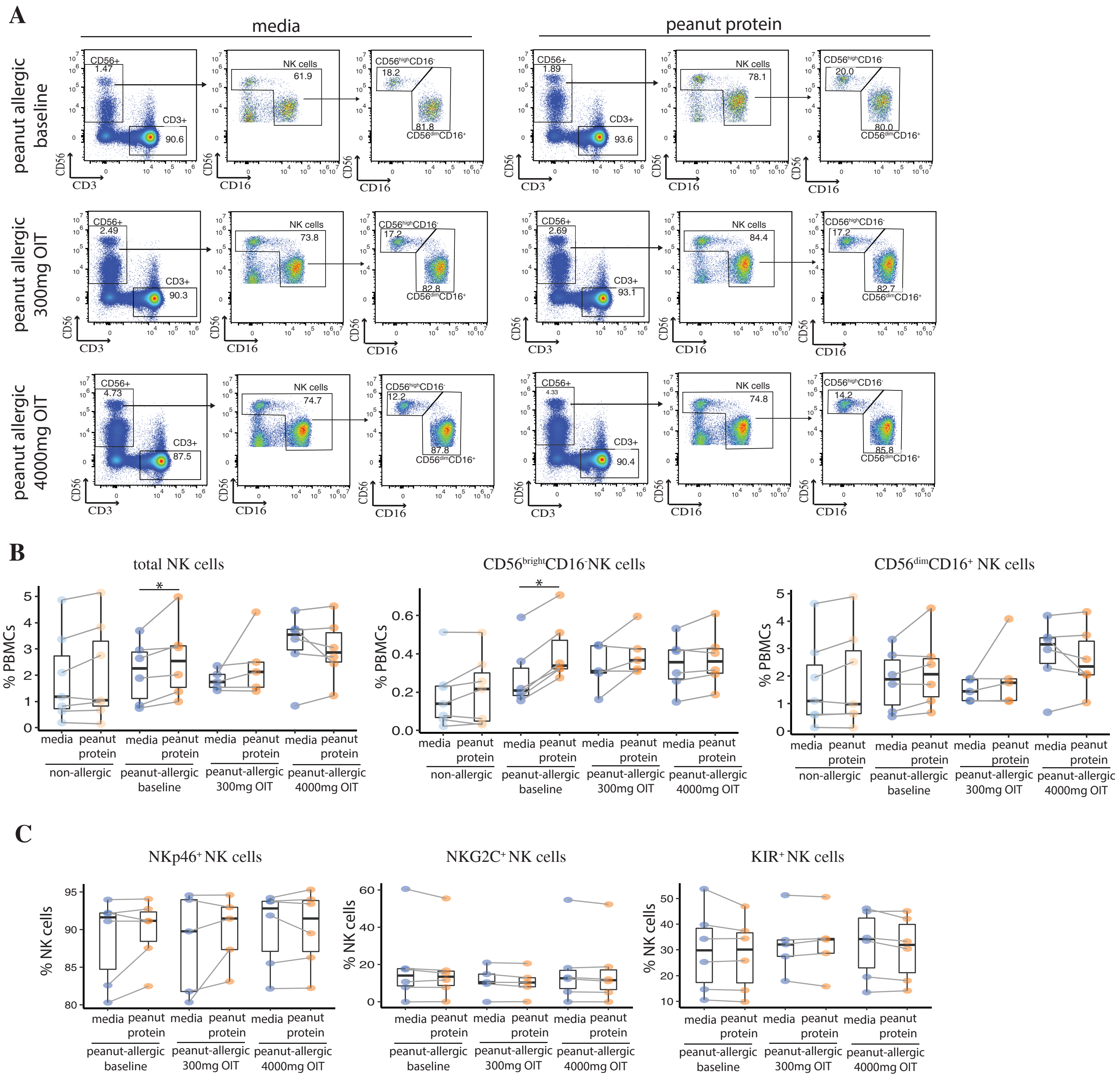

Figure S12. OIT is associated with a decreased expansion of NK cells after peanut stimulation.

(A) Representative flow cytometry plots showing the effect of OIT on total NK, $CD56^{dim}CD16^{+}$ NK and $CD56^{+}CD16^{-}$ NK for 1 PA participant. **(B)** Percentage of total NK, $CD56^{dim}CD16^{+}$ NK and $CD56^{+}CD16^{-}$ NK in PBMCs shown for 6 PA participants before and during OIT. Since the PBMCs sample from one of 6 PA subjects at 300mg peanut OIT was not available, we only have 5 readouts at this timepoint. **(C)** Percentage of $NKp46^{+}$ cells, $NKG2C^{+}$ cells and KIR^{+} cells in total NK cells shown for 6 PA participants before and during OIT. Each pair of points connected by a line represents one subject. Paired sample sets were analyzed using the Wilcoxon signed rank test (two sided). Unpaired sample sets were analyzed using the Wilcoxon rank sum test (two sided). *$P<0.05$.
